# Docosahexaenoic acid inhibits both NLRP3 inflammasome assembly and JNK-mediated mature IL-1β secretion in 5-fluorouracil-treated MDSC: implication in cancer treatment

**DOI:** 10.1038/s41419-019-1723-x

**Published:** 2019-06-19

**Authors:** Adélie Dumont, Charlotte de Rosny, Trinh-Le-Vi Kieu, Sabrina Perrey, Hélène Berger, Aurélie Fluckiger, Tania Muller, Jean-Paul Pais de Barros, Laurent Pichon, Aziz Hichami, Charles Thomas, Cédric Rébé, François Ghiringhelli, Mickaël Rialland

**Affiliations:** 1 Institut National de la Santé et de la Recherche Médicale (INSERM) UMR 1231, Dijon, 21000 France; 2 UFR Sciences de la Vie, Terre et Environnement, Université de Bourgogne Franche-Comté, Dijon, 21000 France; 3 UFR des sciences de santé, Université de Bourgogne Franche-Comté, Dijon, 21000 France; 4 Centre Georges François Leclerc, Dijon, 21000 France

**Keywords:** Cancer, Interleukins

## Abstract

Limitation of 5-fluorouracil (5-FU) anticancer efficacy is due to IL-1β secretion by myeloid-derived suppressor cells (MDSC), according to a previous pre-clinical report. Release of mature IL-1β is a consequence of 5-FU-mediated NLRP3 activation and subsequent caspase-1 activity in MDSC. IL-1β sustains tumor growth recovery in 5-FU-treated mice. Docosahexaenoic acid (DHA) belongs to omega-3 fatty acid family and harbors both anticancer and anti-inflammatory properties, which could improve 5-FU chemotherapy. Here, we demonstrate that DHA inhibits 5-FU-induced IL-1β secretion and caspase-1 activity in a MDSC cell line (MSC-2). Accordingly, we showed that DHA-enriched diet reduces circulating IL-1β concentration and tumor recurrence in 5-FU-treated tumor-bearing mice. Treatment with 5-FU led to JNK activation through ROS production in MDSC. JNK inhibitor SP600125 as well as DHA-mediated JNK inactivation decreased IL-1β secretion. The repression of 5-FU-induced caspase-1 activity by DHA supplementation is partially due to β-arrestin-2-dependent inhibition of NLRP3 inflammasome activity but was independent of JNK pathway. Interestingly, we showed that DHA, through β-arrestin-2-mediated inhibition of JNK pathway, reduces V5-tagged mature IL-1β release induced by 5-FU, in MDSC stably overexpressing a V5-tagged mature IL-1β form. Finally, we found a negative correlation between DHA content in plasma and the induction of caspase-1 activity in HLA-DR^−^ CD33^+^ CD15^+^ MDSC of patients treated with 5-FU-based chemotherapy, strongly suggesting that our data are clinical relevant. Together, these data provide new insights on the regulation of IL-1β secretion by DHA and on its potential benefit in 5-FU-based chemotherapy.

## Introduction

In solid cancers, including colorectal (CRC), pancreatic, gastric, breast, and ovarian cancers, the antimetabolite 5-Fluorouracil (5-FU) represents one of the most commonly used chemotherapeutic drug^1^. Although the 5-FU-based regimen improves the overall survival of patients, the effect of chemotherapeutic cures remains modest in long-term survival, for instance in patients with metastatic CRC^2^. A reason for a limited action of 5-FU-based chemotherapy is a 5-FU-mediated cancer cell death resistance related to overexpression of proteins such as thymidylate synthase (TS)^3^ or lysophosphatidylcholine acyltransferase-2^4^. 5-FU plays an ambivalent role on myeloid-derived suppressor cells (MDSC), which impair innate and adaptive antitumor immune functions^5^. In mice, MDSC are characterized by the expression of CD11b^+^ Gr-1^+^ and consist of polymorphonuclear (PMN-MDSC) and monocytic MDSC (M-MDSC) subpopulations. In humans, MDSC could be minimally defined as M-MDSC with CD11b^+^, CD15^−^, CD33^+^, HLA-DR^neg/low^, CD14^+^ markers and PMN-MDSC with CD11b^+^, CD15^+^, CD33^+^, HLA-DR^neg/low^, CD14^−^ markers^5^. The dual role of 5-FU on MDSC comes from its ability on the one hand to eradicate the pro-tumoral myeloid cells and alleviate anticancer immune suppression^6^ and on the other hand to trigger IL-1β secretion sustaining tumor growth^7^. The 5-FU-induced IL-1β release in MDSC involves activation of nucleotide-binding domain leucin-rich repeat pyrin domain containing receptor 3 (NLRP3) inflammasome by cathepsin-B after lysosomal permeabilization. Secretion of IL-1β triggers Th17 differentiation and production of IL-17A which enhances neoangiogenesis and tumor recurrence^7^.

NLRP3 inflammasome is a multiprotein complex which is able to sense pathogen-associated molecular patterns produced by invading pathogens and damage-associated molecular patterns (DAMPs) generated through endogenous stress conditions^8^. It is accepted that two signals are required for NLRP3 inflammasome activation and formation. The first priming signal induces NF-κB-dependent transcription of IL-1β, IL-18, and NLRP3 genes and makes them available for NLRP3 inflammasome action. The second signal triggered by extracellular stimuli (i.e., microbial components, ATP) leads to induction of various molecular mechanisms including lysosomal permeability, potassium efflux, mitochondrial ROS production, which promote NLRP3 oligomerization and formation of NLRP3 inflammasome complex. The assembly needs interaction of NLRP3 with the adapter ASC (apoptosis-associated speck-like protein containing CARD) recruiting and activating the pro-caspase-1. After self-cleavage, active caspase-1 induces processing of pro-IL-1β and pro-IL-18 into bioactive IL-1β and IL-18, secreted then through nonclassical pathways since they lack a secretory leader sequence^9^.

Omega-3 fatty acids (ω-3 FAs) consumption has clinically proven health benefits in cancer prevention and management^10,11^. Docosahexaenoic acid (DHA) is among the most biologically active members of ω-3 FA with anticancer, anti-inflammatory and immune modulatory properties^12–14^. Thus, DHA-enriched diet is inversely associated with ulcerative colitis and Crohn’s disease and would reduce the risk of colon cancer in patients with these chronic inflammation disorders^15^. DHA supplementation in mouse models of colon cancer also decreases inflammatory status and has antineoplastic action^16–18^. DHA alone or in association with chemotherapeutic agents promotes cancer cell apoptosis and modify cancer neoangiogenesis resulting in an inhibitory effect on tumor growth^12,13,16,19^.

Here, we carried out pre-clinical studies to investigate the ability of DHA to improve 5-FU antineoplastic action, by limiting IL-1β release by MDSC. Moreover, thanks to blood samples from patients submitted to 5-FU-based chemotherapy, we evaluated the correlation between plasma DHA levels and caspase-1 activation in MDSC.

## Materials and methods

### Reagents

DHA (C22:6 n-3), eicosapentaenoic acid (EPA, C20:5 n-3), oleic acid (OA, C18:1 n-9), linoleic acid (LA, C18:2 n-6), SP600125 (JNK inhibitor), N-acetyl-l-Cysteine (NAC), chloroquine (CQ) were purchased from Sigma-Aldrich. TEMPOL was obtained from Abcam (France), Bafilomycine A1 from Enzo (Enzo Life Sciences, France) and 5-FU from ACCORD.

### Patients

Between October 2016 and July 2018, we collected whole blood from metastatic CRC patients (*n* = 46) at the Georges François Leclerc Cancer Center (Dijon, France). mCRC patients were diagnosed in our Cancer Center and received 5-FU-based chemotherapy (5-FU bolus at a dose of 400 mg/m^2^ followed by 5-FU continuous infusion at 2400 mg/m^2^ over 46 h). The 5-FU-based chemotherapy was mostly a combination of 5-FU plus irinotecan or 5-FU plus oxaliplatin. All patients gave an informed consent approved by the local Ethics Committee. The collection of blood samples is performed 24 h after chemotherapy and is authorized by the French authorization (nr. AC2014-2460). No additional blood samples beyond those required for routine testing were taken. Whole blood of mCRC patients was sampled for analysis before (D0) and 24 h after one cycle of 5-FU chemotherapy on heparinized tubes (BD Biosciences).

### FA analysis by gaz chromatography-negative chemical ionization mass spectrometry

Blood samples were collected in patients before and after 5-FU chemotherapy and processed as previously described^20^. Twenty-five microliters of plasma were spiked with 25 µl of SI-Mix solution containing 1300 ng of myristic acid-d3, 5640 ng of palmitic acid-d3, 4200 ng of stearic acid-d3, 3252 ng of LA-d4, 5.2 ng of arachidic acid-d3, 2160 ng of arachidonic acid-d8, 54 ng of behenic acid-d3, 540 ng of DHA-d5, 26 ng of Lignoceric-d4, and 20 ng of cerotic acid-d4 in ethanol. One milliliter of ethanol/butylated hydroxytoluene (50 mg/ml) and 60 µl of potassium hydroxide (10 mol/L) was added to samples before incubation at 56 °C for 45 min under argon atmosphere. Then, aqueous phase extraction was performed with 2 ml of hexane, after addition of hydrochloric acid (1 ml). The upper organic phase was collected after a 10 min centrifugation at 2000*g* and evaporated to dryness under vacuum. In a chemical hood, we dispensed sequentially 5 μl of pentafluorobenzyl bromide (PFB), 90 μl of acetonitrile, and 5 μl of diisopropylbenzylamine before incubation at 37 °C for 30 min and extraction of the resulting PFB-FAs with 1 ml of water and 2 ml of hexane. We evaporated to dryness under vacuum and solubilized PFB-FAs with 100 μl of hexane. We injected 1 μl of PFB-FAs in pulsed split mode on the 7890A gas chromatograph using the HP-5MS fused silica capillary column. Gaz chromatography-mass spectrometry conditions were carrier gas, helium at a flow-rate of 1.1 ml/min; injector temperature set up at 250 °C, pulsed split 10; oven temperature set up at 140 °C, increased of 5 °C/min to 300 °C, and held for 10 min. The mass spectrometer operates under negative chemical ionization mode with methane as reactant gaz. Ion source and quadrupole temperatures were set up at 150 °C. FAs were quantified by calculating their relative response ratios to their closest internal standard. We used an Agilent 7890A gas chromatograph equipped with a 7683 injector and a 5975C Mass Selective Detector (Agilent Technologies) and a GC column HP-5MS fused silica capillary column (30 m × 0.25 mm inner diameter, 0.25 μm film thickness, Agilent Technologies).

### Tumor growth, diet, and 5-FU treatment

All studies with mice were conducted in accordance with the local guidelines for animal experimentation. Protocol no. 8821 was approved by the institutional animal care and use committee of Université de Bourgogne-Franche-Comté. To induce tumor formation, 10^6^ EL4 cells were subcutaneously injected into female C57BL/6J mice (7–9 weeks) from Charles River Laboratories (France). Once tumors were measurable, EL4 tumor-bearing mice were randomly assigned to either a group of mice daily fed an isocaloric control diet with sunflower oil or to a 3% DHA-enriched diet (Omegavie DHA90 TG, Polaris Nutritional Lipids, France). After 1 week of experimental diet, mice received a single intraperitoneal injection of 5-FU at 50 mg per kg body weight (tumor size ~100 mm^2^). The mice were fed experimental diets until the end of the experiment. Tumor growth was monitored over the time and tumor surface was calculated according to the formula: length × width.

### Cell culture and treatments

MSC-2 cell line is CD11b^+^/Gr-1^+^ immortalized MDSC obtained from CT-26 tumor-bearing BALB/c mice^21^.

EL4 thymoma cells and MSC-2 cells were cultured at 37 °C under 5% CO_2_ in RPMI 1640 with 10% (v/v) heat inactivated fetal bovine serum penicillin, streptomycin, amphotericin B antibiotic cocktail, all from Dutscher (Dutscher, Brumath, France). Cell lines were authenticated by examination of morphology and growth characteristics and confirmed to be mycoplasma free. MSC-2 cells were treated with FAs (20–60 µM) bound to FA free-bovine serum albumin (ratio 4:1) (BSA, Sigma-Aldrich) 3 h prior to 5-FU treatment at 1 µM or lipopolysaccharide (LPS) (O55:B5, Sigma-Aldrich) at 100 ng/ml.

### Generation of stable MSC-2 cell line overexpression of V5-tagged mature IL-1β

The coding sequence of mature murine IL-1β with a C-terminal V5 tag was cloned in retroviral construct pMSCV^22^. The E-Platinum retroviral packaging cell line was transfected by Lipofectamine 2000 (Invitrogen) with pMSCV plasmid encoding V5-tagged mature IL-1β for retrovirus production. MSC-2 cells were transduced with cell-free retrovirus solution in the presence of 8 µg/mL of hexadimethrine bromide (Sigma-Aldrich) for 2 days. Stably overexpressing V5-tagged mature IL-1β MSC-2 cells were selected with 7.5 µg/mL of blasticidin (Invivogen).

### Knockdown of ARRB2 expression in MSC-2 cell line

ARRB2 (MISSION shRNA, Sigma-Aldrich) or control shRNA lentivirus vector were co-transfected with the VSV-G envelope and PAX2 packaging plasmids (Addgene) into HEK293T with lipofectamine 2000 (Invitrogen, Fisher scientific). Lentivirus-containing supernatants were collected after 48 h of transfection and filtered. MSC-2 cell lines were transduced by lentiviral particles in the presence of 8 µg/mL of hexadimethrine bromide (Sigma-Aldrich) for 48 h. Selection of stable shRNA ctrl and ARRB2 cell lines was performed with 10 µg/mL of puromycin (ACROS Organics, Fisher scientific).

### Enzyme-linked immunosorbent assay

IL-1β concentration was measured using Mouse IL-1beta/IL-1F2 DuoSet ELISA Kit (R&D Systems, France) according to manufacturer’s instructions. MSC-2 culture media was collected 24 h after treatment and plasma was sampled 48 h after 5-FU injection in tumor-bearing mice.

### Reverse transcription and quantitative PCR

Total RNA from MSC-2 was extracted with Trizol (Life Technologies) and reverse transcribed with M-MLV reverse transcriptase kit from Applied Biosystem (Life technologies). Real-time quantitative polymerase chain reaction (PCR) was performed with SYBR™ Green PCR Master Mix from Applied Biosystem (Life technologies) using a StepOnePlus™ Real-Time PCR System (Applied Biosystems). The sequence of primers were: *β-actin* F: 5′-ATGGAGGGGAATACAGCCC-3′, R: 5′-TTCTTTGCAGCTCCTTCGTT-3′, *18S* F: 5′-GTAACCCGTTGAACCCCATT-3′, R: 5′-CCATCCAATCGGTAGTAGCG-3′, *IL-1β* F: 5′-GGTCAAAGGTTTGGAAGCAG-3′, R: 5′-TGTGAAATGCCACCTTTTGA-3′. Expression was normalized to β-actin and 18 S. Relative expression of RNA targets was determined using the comparative ΔΔ*C*_t_ method.

### Western-blotting analysis

For whole-protein lysates, MSC-2 were washed with ice-cold PBS and lysed with ice-cold RIPA buffer containing phosphatase and protease inhibitor cocktail (P2850 and P8340, Sigma-Aldrich). We cleared protein lysate at 13,000*g* for 15 min at 4 °C. Total protein lysates (60 µg) were resolved by sodium dodecyl sulphate polyacrylamide gel elactrophoresis (SDS-PAGE) and transferred onto a nitrocellulose membrane.

For supernatant precipitation, MSC-2 cells were treated in Opti-MEM I Reduced Serum Medium (ThermoFisher Scientific) for 24 h. Protein precipitation was performed using cell culture supernatant, methanol, and chloroform (ratio 1:1:0.25, respectively). Thus, one volume of supernatant (500 µl) was mixed with one volume of methanol (500 µl) and one-fourth volume of chloroform (125 µl). The mixture was vortexed and centrifuged at 16,000*g* for 10 min. The aqueous methanol layer was removed from the top of the samples. Proteins remained at the phase boundary between the aqueous methanol layer and the chloroform layer. Two volumes of methanol were added and the mixture was vortexed and centrifuged at 16,000*g* for 10 min. Supernatant was removed without disturbing the pellet, and the pellet was dried under nitrogen gas. To finish, pellet was dissolved in 2× loading buffer, incubated 5 min at room temperature and then 5 min at 100 °C. Total proteins were resolved by SDS-PAGE and transferred onto a nitrocellulose membrane.

After saturation for one hour with 5% BSA in tris-buffered saline (TBS)—0.1% Tween-20, membranes were incubated overnight at 4 °C with the primary antibody diluted in saturation solution (1/1000), washed, incubated with the secondary antibody (1/5000) for 1 h at room temperature and washed again before exposure to ECL (Biorad, France). Images acquisition was performed with ImageChemiDoc^TM^ XRS (Biorad, France) and densitometry analysis with Image Lab^TM^ software 5.1.2 (Biorad, France). The antibodies used are anti-IL-1β (R&D systems, AF-401), anti-NLRP3 (Adipogen Cryo-2), anti-ASC (Cell Signaling Technology, B2W8U), anti-cathepsin B (Santa-Cruz S-12), anti-Caspase-1 (Adipogen, Casper-1), anti-p-JNK (Cell Signaling Technology, 98F2), anti-JNK (Cell Signaling Technology, 56G8), anti-β-actin (Sigma-Aldrich, A1978), anti-V5 tag (Invitrogen), Horseradish peroxidase-conjugated anti-mouse, anti-goat, and anti-rabbit (Cell Signaling Technology).

### Caspase-1 activity

To assess caspase-1 activity in MSC-2, the FAM-YVAD-FMK fluorescent probe from Immunochemistry Technologies (Bio-Rad, Marnes-la-coquette, France) was used according to the manufacturer’s instructions and as previously described^7^.

To assess caspase-1 activity in CD11b^+^ Gr-1^+^ MDSC, spleens were collected from treated tumor-bearing mice 48 h after injection of 5-FU or vehicle. Then, they were manually dissociated and passed through a 70-μm cellular sieve. Red blood cells were lysed (NH_4_Cl 0.83%, KHCO_3_ 0.1%, EDTA 0.1 mM) and cells were saturated by using mouse FcR blocking reagent (Miltenyi Biotec, Paris, France). Gr-1^+^ cells were first purified by using phycoerythrin-Cyanine 7-conjugated anti-Gr-1 (RB6-8C5, Ozyme, France) and anti-Cyanine seven microbeads (Miltenyi Biotec). Then, purified cells were incubated with the FAM-YVAD-FMK fluorescent probe according to the manufacturer’s instructions and MDSC were discriminated by using allophycocyanine (APC)-conjugated anti-CD11b (REA592) from Miltenyi Biotec for 20 min at 4 °C, dead cells were exclude using 7-AAD staining.

To assess caspase-1 activity in MDSC from 5-FU-treated colorectal cancer (CRC) patients, 100 µL of heparinized whole blood obtained before and 24 h after 5-FU treatment were incubated with the FAM-YVAD-FMK fluorescent probe according to the manufacturer’s instructions. Then, cells were stained for 20 min at room temperature with lineage cocktail PE-Vio770-conjugated anti-CD3 (BW264/56), anti-56 (REA196), anti-19 (REA675), and anti-20 (REA780), PE-conjugated anti-CD33 (REA775), Viogreen-conjugated anti-CD15 (VIMC6), APC-Vio770-conjugated anti-CD14 (TÜK4), and APC-conjugated anti-HLA-DR (AC122) antibodies and viobility^TM^ 405/452 fixable dye (Miltenyi Biotec) to exclude dead cells. After surface staining, 2 mL of red blood cells lysis/fixation solution (BD) was added for 10 min, cells were centrifuged (450 g for 10 min) and then resuspended in flow cytometry staining buffer (eBioscience, Fisher scientific, France).

All events were acquired by a BD LSR-II flow cytometer with BD FACSDiva software (BD) and data were analyzed using FlowJo software (Tree Star).

### JNK phosphorylation staining

MDSC (CD11b^+^ Gr-1^+^) from tumor-bearing mice were purified and surface labeled as described above. Cells (MSC-2 and CD11b^+^ Gr-1^+^) were fixed and permeabilized using BD Cytofix/Cytoperm buffer (BD), washed with BD Perm/Wash buffer according manufacturer’s recommendations and saturated with Perm/Wash solution containing 5% BSA. Primary antibody against p-JNK (G-9, Ozyme, France) was incubated 1 h at 4 °C, washed and incubated 45 min at 4 °C with Alexa488-conjugated anti-mouse antibody (A-11001, Life Technologies). MDSC were washed and resuspended in staining buffer (eBioscience, Fisher scientific, France) for flow cytometry analysis. JNK phosphorylation in MDSC was determined by Median of Fluorescence Intensity using FlowJo software (Tree Star).

### ROS detection by flow cytometry

Cells were treated for 8 h with 5-FU (1 µM) alone or in combination with DHA (60 µM) or N-acetyl-l-cysteine (NAC, 10 mM). MSC-2 were washed and incubated for 45 min in phenol red-free RPMI containing 10 µM CM-H2DCFDA (Molecular Probes). The cells were collected and analyzed by flow cytometry. The data were acquired with a BD LSR-II flow cytometer using BD FACSDiva software (BD) and analyzed with FlowJo software (Tree Star).

### Immunofluorescence for ASC speck detection

After treatments, cells were washed with PBS, fixed with 4% paraformaldehyde (PFA) at 4 °C for 10 min, permeabilized with 0.2% Saponin (Sigma Aldrich) and saturated with 3% BSA in PBS for 20 min at room temperature. Samples were incubated overnight at 4 °C with anti-ASC antibody (Cell signaling technologies B2W8U). Cells were washed three times in PBS and then incubated with secondary Alexa568-conjugated anti-rabbit (Invitrogen) for 30 min at room temperature. After three washes in PBS, samples were mounted with a drop of Prolong^TM^ diamond antifade mountant containing DAPI (Molecular Probes). Microscopy images were taken on an Axio Imager 2 (Carl Zeiss Microscopy GmbH, Jena, Germany) equipped with an Apotome.2 module (Carl Zeiss GmbH). Images were acquired using an AxioCam MRm monochrome CCD camera (Carl Zeiss GmbH) with filter sets 43 HE (Rhodamine/Alexa568) and 49 (DAPI).

### Proximity ligation assay

Cells were fixed with 4% PFA at 4 °C for 10 min and incubated in a solution of 3% BSA and 0.2% Saponin (Sigma Aldrich) for 20 min. Samples were incubated overnight at 4 °C with primary antibodies: anti-NLRP3 (Adipogen Cryo-2 or Abcam Ab4207) and anti-IL-1β (R&D systems, AF-401), anti-ASC (Cell Signaling Technology, B2W8U), anti-cathepsin B (Santa-Cruz S-12), anti-Caspase-1 (Adipogen, Casper-1) or anti-β-arrestin-2 (Ozyme, C16D9). Cells were incubated with the appropriate probes (anti-Rabbit PLUS, #DUO92002; anti-Goat MINUS, #DUO92006 or anti-Mouse MINUS, #DUO92004) for one hour at 37 °C. Probes were then ligated for 30 min at 37 °C, washed twice and amplified using the manufacturer’s polymerase for 100 min at 37 °C in the dark. Cover glasses were mounted with a drop of mounting medium containing DAPI (Invitrogen). Microscopy images were taken on an Axio Imager 2 (Carl Zeiss Microscopy GmbH, Jena, Germany) equipped with an Apotome.2 module (Carl Zeiss GmbH). Images were acquired using an AxioCam MRm monochrome CCD camera (Carl Zeiss GmbH) with filter sets 43 HE (Rhodamine/Alexa568) and 49 (DAPI).

### Statistical analysis

Data are presented as mean ± SD or SEM. Statistical analyses were performed using Prism (GraphPad). Data normal distribution was determined using the d’Agostino Pearson test and statistical significance was defined by Kruskal–Wallis with Dunn’s post hoc test or one-way ANOVA with Tukey’s post hoc test. Spearman correlation was calculated between two groups. A value of *p* < 0.05 was considered statistically significant. *p < 0.05; **p < 0.01; ***p < 0.001; ****p < 0.0001; ns nonsignificant.

## Results

### DHA decreases 5-FU-induced IL-1β in MDSC and enhances 5-FU anticancer properties

**Fig. 1:**
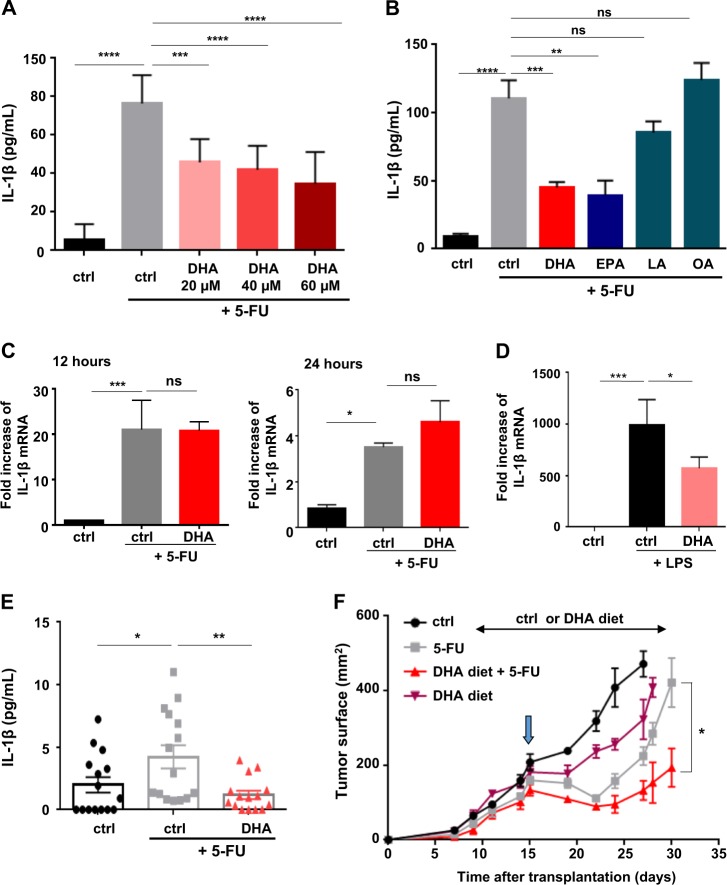
IL-1β secretion is reduced by DHA in 5-FU-treated MDSC. **a** MSC-2 were treated by 5-FU (1 µM) for 24 h ±DHA (20 to 60 µM) and cell culture media were collected for analysis of IL-1β concentration by ELISA. Error bars represent mean ± SD from four independent experiments. **b** Analysis of IL-1β secretion by ELISA in MSC-2 treated with 5-FU (1 µM) for 24 h ±DHA, eicosapentaenoic (EPA), oleic (OA), and linoleic (LA) acids at 60 µM. Error bars represent mean ± SD from 3 to 6 independent experiments. **c** Analysis of IL-1β mRNA expression, by RT-qPCR, in MSC-2 treated for 12 and 24 h with 5-FU ±DHA (60 µM). Error bars represent mean ± SD from three independent experiments. **d** Expression of IL-1β mRNA, by RT-qPCR, in MSC-2 treated for 12 h with 5-FU ±Lipopolysaccharide (LPS). Error bars represent mean ± SD from three independent experiments. **e** Determination of plasma IL-1β content in EL4 tumor-bearing mice fed control (ctrl) or DHA-enriched diet, initiated 7 days prior to a single intraperitoneal injection of 5-FU (50 mg/kg). Plasma was collected 48 h after the injection of 5-FU. **f** Monitoring of tumor growth in mice fed a ctrl diet (*n* = 8, black) or DHA-enriched diet (*n* = 9, purple) and in mice treated with a single 5-FU injection (50 mg/kg; blue arrow) and fed a ctrl diet (*n* = 9, gray) or DHA-enriched diet (*n* = 9, red). Error bars represent mean ± SEM. *p < 0.05; **p < 0.01; ***p < 0.001; ****p < 0.0001; ns nonsignificant

Limitation to 5-FU anticancer action is attributed to the induction of IL-1β release by MDSC. To improve 5-FU efficacy, we evaluated the ability of DHA to reduce IL-1β production in MDSC exposed to 5-FU. We confirmed by ELISA in MSC-2, a murine MDSC cell line, that a 24 h treatment with 5-FU (1 µM) led to an increase in IL-1β secretion (Fig. 1a). Combination of 5-FU treatment with DHA (20–60 µM), inhibited 5-FU induced IL-1β secretion in a dose-dependent manner (Fig. 1a). EPA and DHA belong to ω-3 FA family and both FAs counteract 5-FU action whereas oleic (OA, 60 µM) and LA (60 µM) were not able to reduce IL-1β release (Fig. 1b). Exposure to DHA did not modify 5-FU-mediated cell death rate suggesting that inhibition of 5-FU-induced IL-1β release by DHA is not related to a decrease in IL-1β-producing cells number (Fig. S1a). Moreover, an increase of IL-1β mRNA content in MSC-2 treated for 12 and 24 h with 5-FU (1 µM) was observed (Fig. 1c). In addition, the chemotherapeutic drug also induced TNF-α gene expression (Fig. S1b). We hypothesized that 5-FU activated NF-kB signaling in MDSC and that DHA could counteract the NF-kB activation leading to IL-1β gene-expression repression. Treatment with DHA (60 µM) or with NF-kB signaling inhibitor ACHP (1 µM) for 12 h did not reduce 5-FU-induced IL-1β or TNF-α gene expression in MSC-2 (Fig. 1c and S1b–d). Nonetheless, DHA (60 µM) or ACHP (1 µM) treatments for 12 h were able to decrease LPS-induced IL-1β mRNA expression in MSC-2 (Fig. 1d and S1e). Thus, we showed that NF-kB signaling does not drive IL-1β mRNA expression increase in 5-FU-treated MDSC and that DHA does not inhibit IL-1β secretion through downregulation of 5-FU-induced IL-1β gene expression. Then, DHA efficacy on 5-FU-mediated IL-1β secretion downregulation was investigated in mice bearing EL4 tumors. A single-intraperitoneal injection of 5-FU (50 mg/kg) was performed 15 days after subcutaneous EL4 transplantation in mice fed a standard diet or DHA-enriched diet. A marked increase in plasma IL-1β level of standard-fed mice was observed 48 h following 5-FU treatment as compared to 5-FU nontreated mice (Fig. 1e). Noteworthy, EL4 tumor-mice fed a DHA diet for 7 days prior to 5-FU injection showed a decrease in plasma IL-1β level compared to 5-FU-injected mice fed a standard diet (Fig. 1e). Furthermore, we confirmed that 5-FU therapy eradicates MDSC in tumor-bearing mice^6^, the rate of cell death is not impacted by co-treatment with DHA (Fig. S1f). Moreover, DHA-enriched diet (purple triangles) did not drastically affect tumor growth compared to control diet (ctrl, black circles) (Fig. 1f). By contrast, a single intraperitoneal injection of 5-FU (blue arrow) in EL4 tumor-bearing mice fed either a control (gray squares) or a DHA-enriched diet (red triangles) resulted in a transient tumor regression. Of note, DHA diet improved 5-FU action and significantly limited tumor growth recurrence compared to EL4 tumor-bearing mice fed control diet and treated with 5-FU (Fig. 1f). In addition, we also analyzed in Fig. S1g, the effect of a single intraperitoneal injection of 5-FU (50 mg/kg; blue arrow) in EL4 tumor-bearing mice cotreated with intraperitoneal injections of vehicle (gray squares) or nonesterified DHA (red triangles; 30 µg/g body weight) every 2 days from day 12 (red arrow) till day 32. We showed that injections of nonesterified DHA before and after the single intraperitoneal injection of 5-FU in EL4-bearing mice, promoted a more drastic tumor regression and delayed tumor growth recovery compared to 5-FU-treated mice with intraperitoneal injections of vehicle (Fig. S1g).

Together these data highlighted 5-FU-induced IL-1β secretion downregulation by DHA-treated MDSC and potentiation of 5-FU therapy in DHA-supplemented tumor-bearing mice.

### The inhibition of 5-FU-mediated IL-1β secretion by DHA is dependent on JNK inactivation

**Fig. 2:**
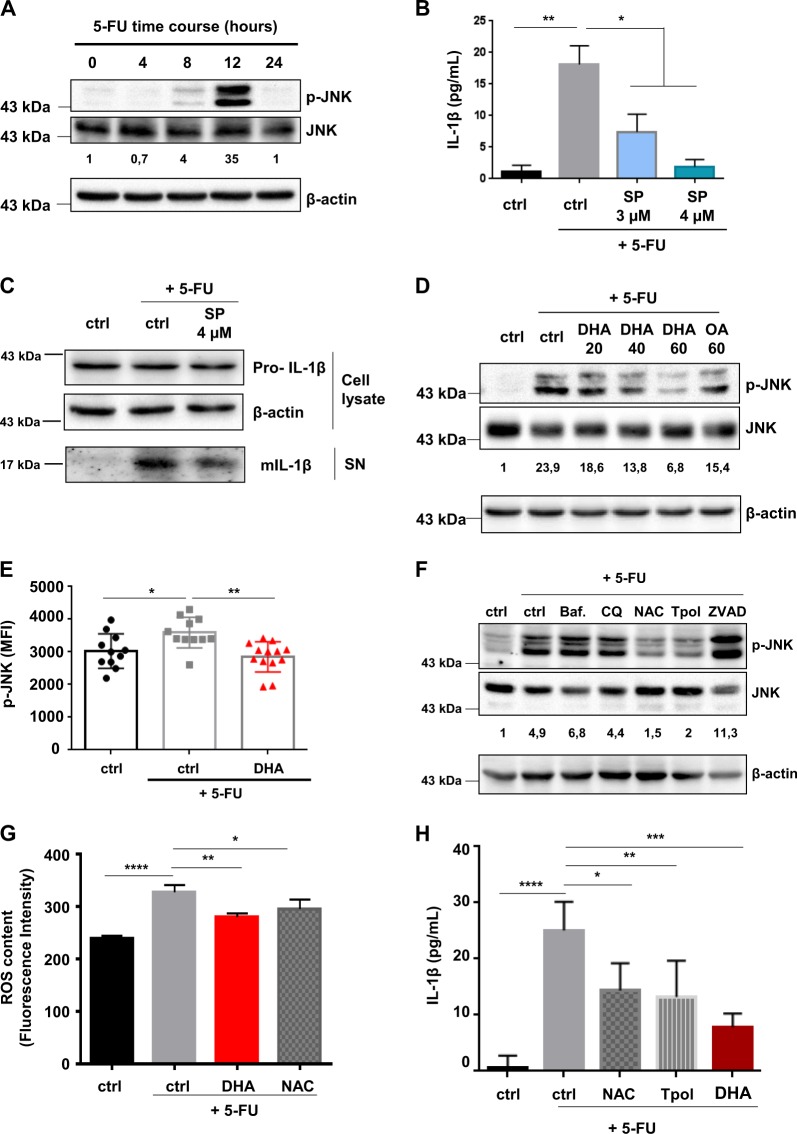
DHA treatment decreases 5-FU-induced JNK activation in MDSC. **a** Representative immunoblotting (*n* = 3) showing expression of phospho-JNK (p-JNK) and total JNK in MSC-2 treated with 5-FU (1 µM) for the indicated times. Quantification of p-JNK and total JNK bands was determined by densitometry. Data are expressed as ratio between p-JNK and total JNK for the different time points and compared to time point (0). **b** Analysis of IL-1β secretion, by ELISA, in MSC-2 treated with 5-FU (1 µM) ±inhibitor of JNK (SP600125; SP) for 24 h. Error bars represent mean ± SD of three independent experiments. **c** Representative immunoblotting (*n* = 4) with anti-IL-1β and anti-β-actin antibodies showing expression of pro-IL-1β in cell lysate and mature IL-1β in supernatant (SN) of MSC-2 treated as in **b**. **d** Representative immunoblotting (*n* = 4) showing phospho-JNK (p-JNK) and total JNK in MSC-2 treated with 5-FU (1 µM) for 12 h ±DHA (20–60 µM) and oleic acid (OA; 60 µM). Relative ratio between p-JNK and total JNK was analyzed by densitometry and compared to the nontreated sample (ctrl). **e** JNK phosphorylation was assessed by flow cytometry in MDSC (CD11b^+^ Gr-1^+^) obtained from spleen of EL4 tumor-bearing mice. Mice were fed a control (ctrl) or DHA-enriched diet initiated 7 days prior to a single intraperitoneal injection of 5-FU (50 mg/kg). MDSC were collected and labeled 24 h after the single 5-FU injection. Error bars represent mean ± SEM. **f** Representative immunoblotting (*n* = 3) showing expression of phospho-JNK and total JNK in 5-FU-treated MSC-2 and co-treated with bafilomycin A (Baf.), chloroquin (CQ), N-acetyl-cysteine (NAC), Tempol (Tpol), and inhibitor of pan-caspases (ZVAD) for 12 h. Ratio between p-JNK and total JNK was determined by densitometry and compared to the nontreated sample (ctrl). **g** Analysis of ROS content. MSC-2 were treated for 8 h with 5-FU (1 µM) ±DHA (60 µM) or anti-oxidant N-acetyl-cysteine (NAC). Cells were loaded with CM-H2DCFDA and analyzed by flow cytometry to determine intracellular ROS content. Error bars represent mean ± SD from three independent experiments. **h** IL-1β secretion by ELISA in MSC-2 treated with 5-FU (1 µM) ±anti-oxidants N-acetyl-cysteine (NAC) and Tempol (Tpol) for 24 h. Error bars represent mean ± SD from three independent experiments. *p < 0.05; **p < 0.01; ***p < 0.001; ****p < 0.0001; ns nonsignificant

JNK pathway is able to regulate IL-1β secretion in inflammatory macrophages through the control of NLRP3 inflammasome activity^23,24^. Then, we evaluated JNK phosphorylation (p-JNK) and activation by 5-FU in MDSC. Increase in p-JNK detection in 5-FU-treated MSC-2 was observed 8 h post-treatment to reach a maximum at 12 h (Fig. 2a). We showed that JNK inactivation with SP600125 (SP) did not affect cell death rate induced by 5-FU (Fig. S2a), but led to a decreased IL-1β release in cell culture medium of MSC-2 treated for 24 h with 5-FU (Fig. 2b, c). Although the chemotherapeutic agent induced both IL-1β and TNF-α gene expression, JNK inactivation by SP600125 was not able to suppress the induction in 5-FU-treated MSC-2 (Fig. S2b and 2c). We investigated the effect of DHA treatment on 5-FU-mediated JNK activation and found that DHA inhibited 5-FU-induced JNK phosphorylation in a dose-dependent manner whereas OA treatment was less efficient than DHA at a similar concentration (Fig. 2d). We confirmed, by flow cytometry analysis, that induction of p-JNK in 5-FU-treated MSC-2 is prevented by DHA (Fig. S2d). Moreover, we showed that 5-FU treatment induced an increase of p-JNK in splenic CD11b^+^ Gr-1^+^ MDSC, obtained from EL4 tumor-bearing mice fed a control diet, whereas DHA-enriched diet significantly reduced 5-FU-induced p-JNK level in these cells (Fig. 2e and Fig. S2e).

We hypothesized that 5-FU treatment might lead to JNK phosphorylation through reactive oxygen species (ROS) formation, lysosomal pathway, or caspase activation. Inhibitors of lysosomal activity (Bafilomycin A1 and Chloroquin) and caspase inhibitor ZVAD did not impact p-JNK level in MSC-2 exposed to 5-FU (Fig. 2f). In contrast, the ROS scavengers N-acetyl-cysteine (NAC) and Tempol (Tpol) were both able to reduce p-JNK (Fig. 2f). Furthermore, we monitored ROS production using the CM-H2DCFDA probe in 5-FU-treated MSC-2. 5-FU induced ROS generation after 8 h of treatment, whereas co-treatment with DHA inhibited 5-FU-mediated ROS production (Fig. 2g). Accordingly, IL-1β secretion induced by 5-FU was repressed by NAC and Tempol in MSC-2 (Fig. 2h).

Our results showed that 5-FU-mediated IL-1β secretion depended on JNK activation and that DHA triggered JNK inactivation through ROS suppression.

### DHA decreases NLRP3 inflammasome assembly and activation in 5-FU-treated MDSC independently of JNK pathway

**Fig. 3:**
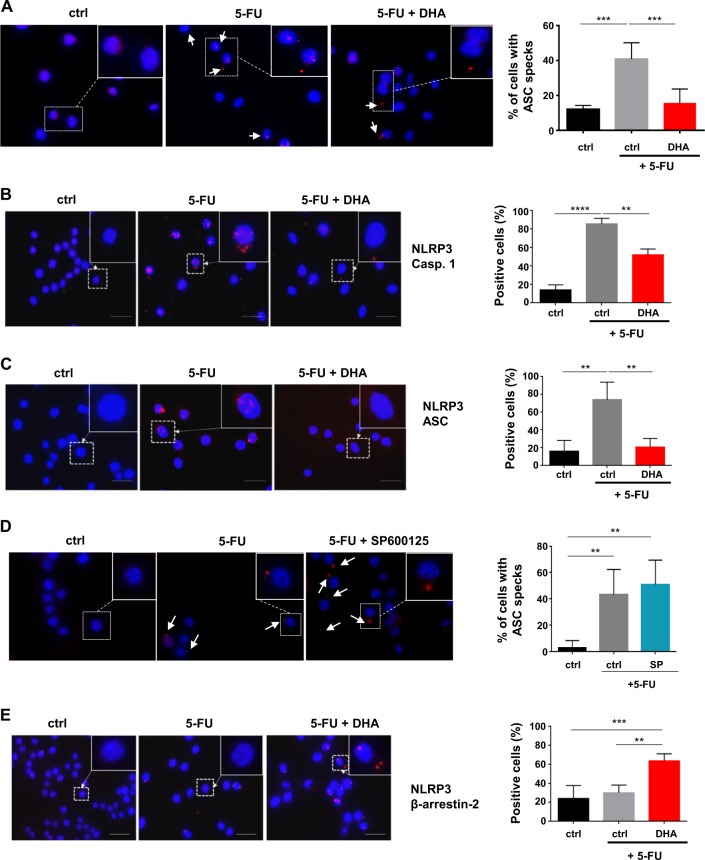
Inhibition of NLRP3 inflammasome assembly and activation by DHA. **a** Analysis of ASC speck formation. MSC-2 were treated for 12 h with 5-FU (1 µM) ±DHA (60 µM) and stained with anti-ASC antibody and DAPI. Arrows show ASC specks. Bar graphs are the mean ± SD of ASC speck-positive cells from three independent replicates. **b**, **c** In situ Proximity Ligation Assay for analysis NLRP3 and caspase-1 **b** or NLRP3 and ASC **c** interaction. MSC-2 were treated for 12 h with 5-FU (1 µM) ±DHA (60 µM). In one experiment, cells (80–100) were analyzed for a positive PLA signal (at least one red dot in a cell). Error bars represent mean ± SD of at least three independent experiments. **d** Analysis of ASC speck formation in MSC-2 treated with 5-FU in combination with JNK inhibitor. Cells were exposed to 5-FU (1 µM) ±SP600125 (4 µM; SP) for 12 h and stained with anti-ASC antibody and DAPI. Arrows show ASC specks. Bar graphs are the mean ± SD of ASC speck-positive cells from two independent experiments. **e** Study NLRP3 and β-arrestin-2 interaction by in situ proximity ligation assay. MSC-2 were treated for 12 h with 5-FU (1 µM) ±DHA (60 µM). As previously, cells were analyzed for a positive-PLA signal (at least one red dot in a cell). Error bars represent mean ± SD of at least three independent experiments. **p < 0.01; ***p < 0.001; ****p < 0.0001; ns nonsignificant. Scale bar = 10 µm

IL-1β secretion induced by 5-FU treatment in MDSC requires NLRP3 inflammasome formation and activation^7^. MDSC (CD11b^+^ Gr-1^+^) are cells that highly express NLRP3 and pro-IL-1β mRNA, compared to their counterpart Gr-1 negative cells purified from spleen of CT-26 tumor-bearing mice (Fig S3a, b). Nevertheless, NLRP3 and pro-IL-1β mRNA expression is not changed in CD11b^+^ Gr-1^+^ obtained from tumor-free (naive) or CT-26 tumor-bearing mice (Fig S3c, d). MSC-2 cell line has been generated by the immortalization of CD11b^+^ Gr-1^+^ cells from CT-26 tumor-bearing mice^21^ and MSC-2 basally expresses components of the NLRP3 inflammasome (Fig S3e). Of note, MSC-2 treatment with 5-FU (1 µM) for 12 h, compared to a combination of 5-FU and DHA (60 µM) did not significantly modify the expression of NLRP3 inflammasome components (Fig. S3e). We investigated the formation of ASC specks in MSC-2 under the control of 5-FU treatment as a readout of inflammasome activation^25^. 5-FU addition increases the percentage of ASC speck positive cells compared to control cells (Fig. 3a). By contrast, co-treatment with DHA reduces the number ASC speck positive cells and prevents 5-FU-induced NLRP3 inflammasome activation (Fig. 3a). We then investigated the effect of DHA on the spatial rearrangement between NLRP3 protein and the scaffold protein ASC or caspase-1 by in situ proximity ligation assay (PLA)^26^. The number of cells with a positive-PLA signal (at least one red dot in a cell) evidencing NLRP3-ASC or NLRP3-caspase-1 proximity increased in MSC-2 treated with 5-FU (1 µM) for 12 h compared to untreated MSC-2 (Fig. 3b, c). The mean number of red dots per cell also increased in 5-FU-treated MSC-2 (Fig. S3f). By contrast, the addition of DHA in 5-FU-treated MSC-2 inhibited NLRP3 and ASC or NLRP3 and caspase-1 interaction (Fig. 3b, c and S3f). Unexpectedly, JNK inhibitor (SP600125; 4 µM) did not inhibit the formation of ASC specks (Fig. 3d) nor the interaction between NLRP3 and caspase-1 (Fig. S3g) induced by 5-FU treatment. These results exclude a role for the JNK pathway in DHA-mediated inhibition of NLRP3 inflammasome formation and activation. Lysosomal permeabilization mediated by 5-FU controls NLRP3 inflammasome activation through cathepsin-B release and its binding to NLRP3^7^. Thus, we confirmed that 5-FU treatment activates lysosomal permeabilization (Fig. S3h) and increases cell proportion with a positive-PLA signal as well as the mean number of red dots per cell highlighting the interaction between NLRP3 and cathepsin-B (Fig. S3i, j). Noteworthy, the combination with DHA does not repress neither lysosomal permeabilization (Fig. S3h) nor NLRP3 and cathepsin-B interaction (Fig. S3i, j). The protein β-arrestin-2 inhibits IL-1β secretion through its interaction with NLRP3 inflammasome^27^. Therefore, we evaluated the effect of DHA on NLRP3 and β-arrestin-2 interaction. The co-treatment of MSC-2 with 5-FU and DHA increased PLA-positive cells, as compared to control or 5-FU-treated MSC-2. These results suggest that DHA-mediated inhibition of NLRP3 inflammasome assembly could occur through the binding of β-arrestin-2 to NLRP3 protein (Fig. 3e and S3k).

Overall, our results suggested that in MDSC DHA inhibited NLRP3 inflammasome formation induced by 5-FU in β-arrestin-2-dependent manner and independently of cathepsin-B and JNK.

### Inhibition of caspase-1 activity by DHA in 5-FU-treated MDSC is independent of JNK pathway

**Fig. 4:**
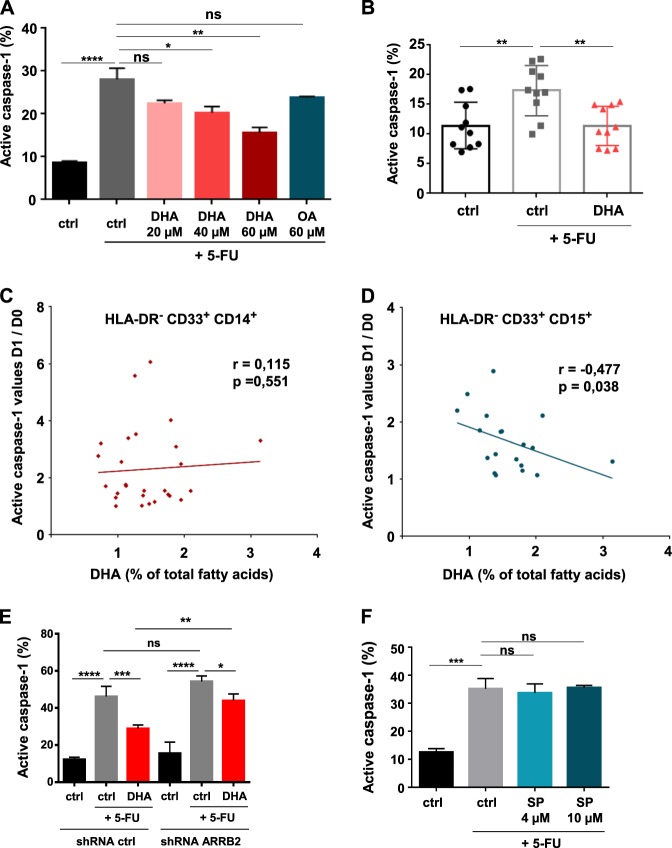
Repression of caspase-1 activity in 5-FU-treated MDSC mediated by DHA exposure. **a** Analysis of 5-FU-induced caspase-1 activity by FLICA in MSC-2 treated with 5-FU (1 µM) for 12 h ±DHA (20–60 µM) or oleic acid (OA). Error bars represent mean ± SD from three independent experiments. **b** Measure of caspase-1 activity by FLICA in MDSC (CD11b^+^ Gr-1^+^) purified 48 h after a single intraperitoneal injection of 5-FU (50 mg/kg) to EL4 tumor-bearing mice fed control (ctrl) or DHA-enriched diet. Error bars represent mean ± SEM. **c**, **d** Correlation between plasma DHA content before 5-FU therapy and ratio of caspase-1 activity (after (D1) and before (D0) 5-FU chemotherapy) in HLA-DR^−^ CD33^+^ CD14^+^ cells **c** or in HLA-DR^−^ CD33^+^ CD15^+^ cells **d** (*n* = 26 patients). **e** Effect of DHA on 5-FU induced caspase-1 activity in β-arrestin-2-silenced MDSC. MSC-2 with stable knockdown of β-arrestin-2 was obtained by transduction with β-arrestin-2 (ARRB2) shRNA lentiviral vector and compared to ctrl shRNA vector. Cells were treated with 5-FU (1 µM) ±DHA (60 µM) for 12 h and harvested for active caspase-1 analysis by FLICA. Error bars represent mean ± SD from three independent experiments. **f** Caspase-1 activity analyzed by FLICA in 5-FU-treated MSC-2 for 12 h ±JNK inhibitor SP600125 (4 and 10 µM). Error bars represent mean ± SD from four independent experiments. *p < 0.05; **p < 0.01; ***p < 0.001; ****p < 0.0001; ns nonsignificant

As aforementioned the role of JNK in DHA-mediated repression of IL-1β release does not involve NLRP3 inflammasome assembly and activation. An additional control of 5-FU-mediated IL-1β secretion by DHA might concern a JNK pathway-dependent regulation of caspase-1 activity within inflammasomes through β-arrestin-2. We found that exposure to 5-FU for 12 h increased the percentage of active caspase-1-positive MSC-2 cells, while combination with DHA limited the effect of 5-FU by reducing the percentage of caspase-1-positive cells in a dose-dependent manner (Fig. 4a). Accordingly, treatment with 5-FU in tumor-bearing mice fed a control diet promoted caspase-1 activation in MDSC (CD11b^+^ Gr-1^+^ cells) purified from spleen. By contrast, MDSC originating from tumor-bearing mice treated by 5-FU and fed a DHA-enriched diet presented a lower caspase-1 activity (Fig. 4b).

We investigated the correlation between the percentage of DHA in total plasma FAs prior to a 5-FU-based chemotherapy (D0) and a change of caspase-1 activity in circulating MDSC purified on D0 compared to those purified one day after chemotherapy (D1), in patients with colorectal cancer (*n* = 46). Correlation between plasma DHA content and caspase-1 activation in HLA-DR^−^ CD33^+^ CD14^+^ and HLA-DR^−^ CD33^+^ CD15^+^ MDSC in patients treated with a 5-FU-containing chemotherapeutic regimen was not significant (data not shown). Focusing on patients with increased post-chemotherapeutic caspase-1 activity (Fig. S4a, b), we showed a negative correlation between plasma DHA content and increase of caspase-1 activity between D0 and D1 in HLA-DR^−^ CD33^+^ CD15^+^ MDSC (Fig. 4c, d).

When investigating β-arrestin-2 and JNK pathway putative role in DHA-mediated inhibition of caspase-1 activity, we showed a role of β-arrestin-2 in the downregulation of 5-FU-activated caspase-1 by DHA. We stably abrogated β-arrestin-2 expression with shRNA in MSC-2 (Fig S4c), and demonstrated that inhibition of 5-FU-induced caspase-1 activation observed in DHA-treated shRNA control (ctrl) MSC-2 was less efficient than in β-arrestin-2-silenced MSC-2 (Fig. 4e). On the other hand, we evidenced that SP600125 (SP) treatments did not decrease caspase-1 activity in 5-FU-treated MSC-2 (Fig. 4f) assessed at 12 h; a time point where p-JNK reached its highest level (Fig. 2a). Thus, JNK inhibition does not mimic DHA effect on caspase-1 activation in 5-FU-treated MDSC. This data evidences that DHA suppresses part of NLRP3 inflammasome activity in 5-FU-treated MDSC through β-arrestin-2 by a JNK-independent mechanism.

### DHA limits JNK-dependent secretion of mature IL-1β in 5-FU-treated MDSC

**Fig. 5:**
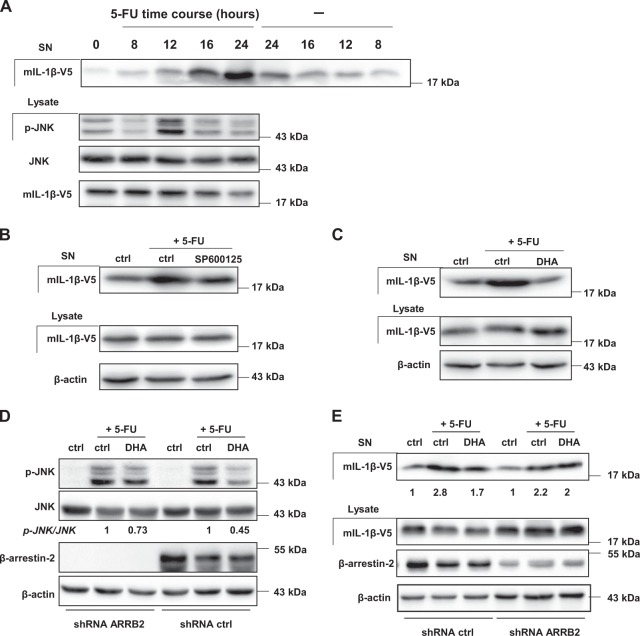
DHA decreases 5-FU-mediated mature IL-1β release. MSC-2 cells were stably transduced with a pMSCV plasmid containing V5-tagged mature IL-1β. **a** Analysis by western-blotting of JNK phosphorylation (p-JNK), total JNK (JNK) and mature V5-tagged IL-1β content with anti-p-JNK, anti-JNK and anti-V5 antibodies in cell lysate and supernatant (SN) of MSC-2. Cells were treated by 5-FU (1 µM) at indicated times. Data represent three replicates. **b** Mature IL-1β level, determined by western-blotting in supernatant (SN) and cell lysates with anti-V5 and anti-β-actin antibodies. MSC-2 were treated for 24 h with 5-FU (1 µM) ±SP600125 (4 µM). Image is representative of four independent experiments. **c** Mature IL-1β level was determined as in **b** after 24 h of treatment with 5-FU (1 µM) ±DHA (60 µM). Data are representative of four independent experiments. **d** Relative changes in p-JNK/JNK ratio were quantified by western-blotting in MSC-2 stably expressing β-arrestin-2 (ARRB2) or ctrl shRNA. MSC-2 were treated with 5-FU (1 µM) ±DHA (60 µM) and were harvested 12 h after the treatment for western-blotting analysis with anti-p-JNK, anti-JNK, anti-β-arrestin-2, and anti-β-actin antibodies. Ratio p-JNK to total JNK is determined by densitometry and the comparison is relative to the 5-FU condition (ctrl). Data are representative of three replicates. **e** Mature IL-1β secretion was evaluated in β-arrestin-2 (ARRB2)-silenced and ctrl shRNA MSC-2 stably expressing V5-tagged mature IL-1β treated for 24 h with 5-FU (1 µM) ±DHA (60 µM). Protein levels from supernatants (SN) and cell lysates were analyzed by western-blotting with anti-V5, anti-β-arrestin-2, and anti-β-actin antibodies. Relative change of V5-tagged mature IL-1β secretion was determined by densitometry in MSC-2 treated with 5-FU ±DHA compared to ctrl MSC-2. Image is representative of three independent experiments

To further define JNK pathway function in IL-1β release regulation, we established a MSC-2 cell line that stably expressed the V5-tagged mature IL-1β form (mIL-1β-V5) (Fig. S5a). Such cell line allows the evaluation JNK pathway role on DHA-mediated inhibition of mature IL-1β independently of pro-form IL-1β processing. We confirmed in MSC-2 overexpressing mIL-1β-V5 that JNK phosphorylation (p-JNK) is observed 12 h post-treatment with 5-FU (Fig. 5a and Fig. S5b). Accordingly, 5-FU-mediated mIL-1β-V5 release is detected after 16 h of treatment (Fig. 5a). Then, we analyzed the effect of a JNK inhibitor on 5-FU-induced mIL-1β-V5 secretion at 24 h and found that SP600125 limits mature IL-1β release triggered by 5-FU (Fig. 5b). The inhibition of mature IL-1β release by SP600125 does not rely on a change in 5-FU-mediated cell death (Fig. S5c). Then, we investigated the ability of DHA to regulate mIL-1β-V5 release in 5-FU-treated MSC-2. The exposure to 5-FU for 24 h induced mIL-1β-V5 secretion by MDSC, while its combination with DHA drastically reduced mIL-1β-V5 release without affecting 5-FU-mediated cell death (Fig. 5c and S5d). We explored the effect of DHA on 5-FU-induced p-JNK in ctrl and β-arrestin-2-silenced MSC-2. Although DHA represses induction of 5-FU-mediated p-JNK in shRNA ctrl MSC-2, the lack of β-arrestin-2 expression in shRNA ARRB2 MSC-2 did not allow the inhibition of 5-FU-induced p-JNK by DHA (Fig. 5d). Therefore, we analyzed DHA action on secretion of mature IL-1β-V5 in MSC-2 expressing different level of β-arrestin-2 and treated by 5-FU. We confirmed the data showing that DHA counteracted 5-FU-activated mIL-1β-V5 release in shRNA ctrl MSC-2, whereas DHA effect on 5-FU-induced IL-1β-V5 secretion is lost in β-arrestin-2-silenced MSC-2 (Fig. 5e).

Altogether, our data show that DHA controls mature IL-1β release via JNK inactivation and requires β-arrestin-2 for such inactivation.

## Discussion

IL-1β production contributes to cancer development and progression but also limits the 5-FU anticancer effect through NLRP3 inflammasome activation in MDSC^7,28–30^. In NLRP3^−/−^ and IL-1β^−/−^ mice models, treatment with 5-FU shows a long term tumor growth regression^7^. Therefore, inhibition of NLRP3 inflammasome activity and/or IL-1β secretion might be an approach to correct 5-FU ambivalent action in cancer patients. A recent phase II trial (IRAFU) suggested that the use of IL-1 receptor antagonist (Anakinra) together with 5-FU and bevacizumab (anti-VEGF) increased progression-free survival and overall survival in mCRC patients^31^. However, 78% of patients showed therapy-related grade 3 toxicity^31^. By contrast, omega-3 FAs such as DHA found in fish oils potentiated 5-FU-based therapy and reduced chemotherapy toxicities according to both epidemiological and preclinical studies^32–38^. Nevertheless, the quality of fish oils seems to be important for a positive effect in combination with chemotherapeutic drugs since hexadeca-4,7,10,13-tetraenoic acid, a minor FA found in fish oils, would limit efficacy of anticancer drugs such as 5-FU^39,40^.

**Fig. 6:**
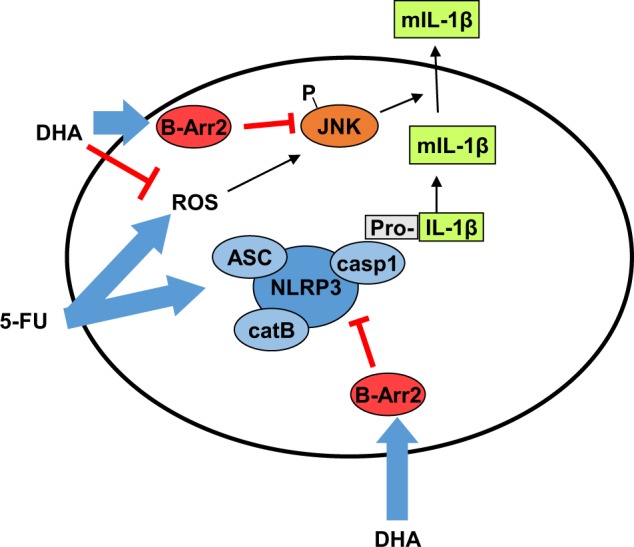
Graphical abstract. DHA limits 5-FU-induced IL-1β release in MDSC through inhibition of NLRP3 inflammasome assembly and JNK-dependent mature IL-1β secretion

We demonstrated that highly purified DHA enhances 5-FU anti-tumor efficacy in a murine cancer model, by repressing 5-FU-induced IL-1β secretion in MDSC under the control of NLRP3 inflammasome and JNK pathway through β-arrestin-2 (Fig. 6).

Inflammasome priming is the first step on IL-1β secretion regulation and takes place through IL-1β and NLRP3 mRNA expression induction in activated macrophages^41^. We showed that DHA, as in macrophages^42^, inhibits NF-κB-dependent IL-1β expression in LPS-activated MDSC. Addition of 5-FU to MDSC acts as a priming signal and induces IL-1β mRNA expression. Nevertheless, 5-FU-activated pathway in MDSC is different from LPS induced pathway, since IL-1β mRNA expression is not suppressed by neither DHA nor by an NF-κB signaling inhibitor (ACHP). Furthermore, the fact that DHA limits 5-FU-mediated ROS generation without affecting IL-1β mRNA expression, excluded 5-FU-induced NLRP3 inflammasome priming regulation by ROS, which has been described for LPS-activated macrophages^43^. In this context, DHA counteracts ROS production whereas it induces ROS in cancer cells^44^.

Maturation of IL-1β in 5-FU-treated MDSC requires caspase-1 activation subsequently to NLRP3 inflammasome formation^7^. We highlighted that DHA inhibits chemotherapeutic-induced NLRP3 inflammasome assembly and activation evidenced by an impairment of NLRP3 and ASC interaction, leading to a decrease in ASC speck formation and caspase-1 activity in MDSC. Regulation of caspase-1 activity has already been described in DHA-treated macrophages exposed to LPS and nigericin^27^. Activation of GPR120 and GPR40 by DHA induces the binding of β-arrestin-2 to NLRP3 inflammasome, repressing caspase-1 activity^27^. GPR120 is expressed in MSC-2 (data not shown) and we demonstrated that DHA treatment increases the interaction between β-arrestin-2 and NLRP3 in 5-FU-treated MDSC. Furthermore, β-arrestin-2 silencing partially abrogates DHA ability to decrease 5-FU-induced caspase-1 activation in MDSC. Therefore, the interaction between NLRP3 and β-arrestin-2 triggered by DHA inhibits 5-FU-induced caspase-1 activation in MDSC.

In patients with mCRC, 5-FU-based chemotherapy induced caspase-1 activity in both HLA-DR^−^ CD33^+^ CD14^+^ and HLA-DR^−^ CD33^+^ CD15^+^. Nevertheless, an interpatient variability of 5-FU-mediated caspase-1 activation in MDSC is observed and could depend on 5-FU metabolism, which involves a competition between elimination of the drug by dihydropyrimidine dehydrogenase (DPD) and formation of active metabolite targeting the TS. A variation of DPD expression between patients is described and correlated with 5-FU efficacy and toxicity^45^. Moreover, an inverse correlation between 5-FU-induced caspase-1 activation and TS expression in MDSC from patients has been reported^7^. Therefore, the negative correlation between circulating DHA content and postchemotherapeutic caspase-1 activation in HLA-DR^−^ CD33^+^ CD15^+^ and not in HLA-DR^−^ CD33^+^ CD14^+^ MDSC could originate from a different ability of DHA to regulate TS or DPD expression in human MDSC subpopulations. In addition, a different expression pattern of GPR120 or β-arrestin-2 in MDSC subpopulations could explain the specific ability of DHA to inhibit 5-FU-induced caspase-1 activation in HLA-DR^−^ CD33^+^ CD15^+^.

Interestingly, we showed that inhibition of 5-FU-mediated JNK pathway activation by SP600125 blocked IL-1β release. In inflammasome-activated macrophages, JNK phosphorylates NLRP3 and ASC leading to NLRP3 inflammasome activation and IL-1β secretion^23,24^. However, JNK inactivation in 5-FU-treated MDSC did not appear to regulate NLRP3 inflammasome activity, since its inhibition did not repress ASC speck formation and caspase-1 activity. Nevertheless, JNK pathway inactivation by DHA or SP600125 inhibits the mature IL-1β secretion step independently of cell membrane rupture. Indeed, we observed that SP600125 and DHA did not change the rate of cell death induced by 5-FU. Regulation of IL-1β release is a complex process and deserves further investigations. A critical point would be the change of membrane permeability under the control of JNK and might involve gasdermin D, which once activated by caspase-1, contributes to IL-1β secretion^22^. Although ROS inhibition did not repress 5-FU-mediated caspase-1 activity in MDSC^7^, we showed here that IL-1β secretion is regulated by 5-FU-generated ROS leading to activation of JNK independently of lysosomal and caspase activity. The blockade of 5-FU-mediated JNK activation by DHA originates from a direct inhibition of ROS production suppressing mature IL-1β secretion. In addition, we found that β-arrestin-2 is required for DHA-mediated inhibition of JNK activation and mature IL-1β release induced by 5-FU-treated MDSC. Binding of β-arrestin-2 onto TAB1 is able to inhibit JNK activation induced by TAK1 in LPS-treated macrophages^46^ and might represent a mechanism for the regulation of JNK-dependent IL-1β secretion by DHA.

Altogether, our work suggests that DHA might have the potential to improve 5-FU anti-cancer effect.

## Supplementary information

Supplemental information

Supplemental Figure 1

Supplemental Figure 2

Supplemental Figure 3

Supplemental Figure 4

Supplemental Figure 5
