# Supplementary material for: Docosahexaenoic acid inhibits both NLRP3 inflammasome assembly and JNK-mediated mature IL-1β secretion in 5-fluorouracil-treated MDSC: implication in cancer treatment
Source: Cell Death Dis. 2019 Jun 19;10(7):485. doi: 10.1038/s41419-019-1723-x (PMC6584690; doi:10.1038/s41419-019-1723-x)
Supplement: Supplementary file 1 — Supplemental information [file 41419_2019_1723_MOESM1_ESM.doc]

Docosahexaenoic acid inhibits both NLRP3 inflammasome assembly and JNK-mediated mature IL‑1β secretion in 5‑Fluorouracil-treated MDSC: implication in cancer treatment

**Supplemental Figure Legends**

Fig. S1

**a** Viability of MSC-2 treated for 12 and 24 hours with 5‑FU +/- DHA (docosahexaenoic acid) or OA (oleic acid) was assessed using 7-AAD staining. Cells were analyzed by flow cytometry. Error bars represent mean±SD from four independent experiments. **b** Analysis of TNF-α mRNA expression by RT-qPCR in MSC-2 treated for 12 hours with 5‑FU (1 µM) +/- DHA (60 µM). Error bars represent mean±SD from three independent experiments. **c-d** IL-1β (c) and TNF-α (d) mRNA expression by RT-qPCR in MSC-2 treated for 12 hours with 5‑FU (1 µM) +/- NF‑kB inhibitor (ACHP, 1 µM). Error bars represent mean±SD from three independent experiments. **e** IL-1β mRNA expression analysis by RT-qPCR in MSC-2 treated for 12 hours with LPS (100 ng/ml) +/- NF‑kB inhibitor (ACHP, 1 µM). Error bars represent mean±SD from three independent experiments. **f** Percentage of CD11b^+^ Gr-1^+^ cells was determined in spleens of EL4 tumor‑bearing mice fed control (ctrl) or DHA diet initiated 7 days prior to 5‑FU injection (50 mg/kg). Cells were collected 48 hours after 5‑FU injection. Error bars represent mean±D from four independent experiments. **g** Effect of non‑esterified DHA injections in EL4 tumor‑bearing mice treated by 5‑FU. EL4 tumor growth monitoring was performed in two groups of mice composed of 5‑FU‑non‑treated mice (ctrl; black circles, n=4) and mice treated by a single intraperitoneal injection of 5-FU (50 mg/kg; gray squares and red triangles). The group of 5‑FU‑treated mice received intraperitoneal injections of vehicle (gray squares, 5‑FU+veh.; n=7) or free DHA at 30 µg/g body weight (red triangles, 5‑FU+DHA; n=7) every two days until the end of tumor

growth monitoring. The first free DHA injection (red arrow) started 6 days before the single intraperitoneal injection of 5‑FU (blue arrow).

*p< 0.05; **p<0.01; ***p<0.001; ns, non-significant.

Fig. S2

**a** Viability of MSC-2 treated by 5-FU (1 µM) +/- SP600125 for 24 hours analyzed by 7‑AAD staining in flow cytometry. Error bars represent mean±SD from three independent experiments. **b** IL-1β mRNA expression analysis in MSC-2 treated for 12 and 24 hours with 5‑FU +/- SP600125 (SP; 4 µM). Error bars represent mean ± SD from three independent experiments. **c** Analysis of TNF-α mRNA expression in MSC-2 treated for 12 hours with 5‑FU +/- SP600125 (SP; 4 µM). Error bars represent mean±SD from three independent experiments. **d-e** Flow cytometry analysis of JNK phosphorylation (p‑JNK) in MDSC. Cells were labelled with isotype control or anti-p-JNK (G9) antibodies and with Alexa488‑conjugated anti‑mouse (A-11001) antibody. MSC‑2 were treated for 12 hours with 5‑FU +/- DHA (60 µM). Isotype control was performed on 5‑FU-treated MSC‑2. Cytogram profiles are representative of three independent experiments (d). MDSC (CD11b^+^ Gr-1^+^) were purified from spleens of EL4 tumor‑bearing mice. Mice are divided into a group fed control diet without 5‑FU injection (ctrl) or with a single 5‑FU injection (5‑FU) and fed a DHA-enriched diet initiated 7 days before a single 5‑FU injection (5‑FU+DHA). MDSC were harvested 24 hours after 5‑FU injection. MDSC from 5‑FU‑treated mice were used for isotype control (e). *p< 0.05; **p<0.01; ns, non-significant.

Fig. S3

**a-b** Relative expression of IL-1β and NLRP3 mRNA analyzed by RT-qPCR in CD11b^+^ Gr‑1^+^ (MDSC) purified from spleens of CT-26 tumor-bearing mice (n=14). Expression was normalized to β-actin and 18S. **c-d** Relative expression of IL-1β and NLRP3 mRNA analyzed by RT-qPCR in Gr-1 negative cells (Gr-1 neg.) and CD11b^+^ Gr‑1^+^ cells purified from spleens of tumor-free (naïve, n=6) or CT-26 tumor-bearing (n=14) mice. Expression was normalized to β-actin and 18S. **e** Expression of NLRP3 inflammasome components analyzed by western‑blotting with antibodies raised against NLRP3, caspase‑1, IL-1β, ASC, cathepsin‑B and β-actin. MSC‑2 protein lysates were collected 12 hours after treatment with 5‑FU (1 µM) +/- DHA (20 to 60 µM) or oleic acid (OA). Representative image of two independent experiments. **f** Analysis of proximity between NLRP3 and caspase-1 or ASC has been performed by *in situ* Proximity Ligation Assay in MSC‑2 treated with 5-FU +/- DHA for 12 hours. The mean of red dot numbers per cell was determined with ICY software. Error bars represent mean±SD from at least three independent experiments. **g** *In situ* Proximity Ligation Assay for analysis of interaction between NLRP3 and caspase-1 in MSC‑2 treated for 12 hours with 5‑FU (1 µM) +/-SP600125 (SP; 4 µM). In one experiment, cells (80-100) were analyzed for a positive PLA signal (at least one red dot in a cell). Bar graphs are the mean ±SD of three independent experiments. **h** Lysosomal permeability using Lysotracker Deep Red labelling has been evaluated in 5‑FU‑treated MSC‑2 cells +/- DHA (60µM) or bafilomycine A1 (Baf). Error bars represent mean ± SD from three independent experiments. **i-j** Analysis of proximity between NLRP3 and cathepsin-B has been performed by *in situ* Proximity Ligation Assay in MSC‑2 treated with 5-FU +/- DHA (60 µM) for 12 hours. In one experiment, cells (80-100) were analyzed for a positive PLA signal (at least one red dot in a cell) and the mean of reds dot number per cell was determined with ICY software. Error bars represent mean±SD from at least three independent experiments. **k** Analysis of NLRP3 and β‑arrestin‑2 interaction was determined by *in situ* Proximity Ligation Assay in MSC‑2 treated with 5-FU +/- DHA for 12 hours. The mean of red dot number per cell was determined with ICY software. Error bars represent mean±SD from at least three independent experiments. *p< 0.05; **p<0.01; ***p<0.001; ****p<0.0001; ns, non-significant.

Fig. S4

**a-b** Analysis of active caspase-1 by FLICA in HLA-DR^-^ CD33^+^ CD14^+^ cells (**a**) or in HLA‑DR^-^ CD33^+^ CD15^+^ cells (**b**) from patients with metastatic CRC (n=46) before (D0) and after (D1) 5‑FU‑based chemotherapy. Panels in red (**a**) and blue (**b**) showed patients with increase caspase-1 activity (D1/D0) in HLA-DR^-^ CD33^+^ CD14^+^ cells or in HLA-DR^-^ CD33^+^ CD15^+^ cells. **c** Stable extinction of β‑arrestin‑2 expression in MSC-2. Cells were infected by β‑arrestin‑2 (ARRB2) or control (ctrl) shRNA lentiviruses and selected with puromycin. Protein lysates were prepared from MSC‑2 for immunoblotting with anti‑β‑arrestin‑2 and anti‑β‑actin antibodies. Image is representative of two independent experiments.

Fig. S5

**a** Analysis of V5‑tagged mature IL-1β form (mIL-1β-V5) expression in nontransfected (-) and stably expressing (mIL-1β-V5) MSC‑2. **b** Induction of JNK phosphorylation by 5‑FU (1 µM for 12 hours) in MSC‑2 stably overexpressing mIL-1β-V5 analyzed by western‑blotting with anti‑p‑JNK, anti‑JNK, anti-V5 and anti-β‑actin antibodies. **c** Viability of mIL-1β-V5-expressing MSC‑2 cells treated for 24 hours with 5‑FU +/- SP600125 (SP; 4 µM) was analyzed after 7‑AAD staining by flow cytometry. **d** Viability of mIL-1β-V5-expressing MSC‑2 treated for 24 hours with 5‑FU +/- DHA (60 µM) analyzed after 7‑AAD staining by flow cytometry. Error bars represent mean±SD from three independent experiments. **p<0.01; ***p<0.001; ****p<0.0001; ns, non-significant.

**Supplemental Material and Methods**

***Treatment of MSC-2 with LPS and NF‑kB inhibitor***

Cells were treated with Lipopolysaccharide (LPS) (O55:B5, Sigma-Aldrich) at 100 ng/mL and NF‑kB inhibitor (ACHP, Tocris) at 1 µM for 12 hours.

***Reverse transcription and quantitative PCR***

Total RNA from MSC-2 and CD11b^+^ Gr-1^+^ purified from spleens of tumor-bearing mice was extracted with Trizol (Life Technologies) and reverse transcribed with M‑MLV reverse transcriptase kit from Applied Biosystem (Life technologies). Real-time RT‑qPCR was performed with SYBR^TM^ Green PCR Master Mix from Applied Biosystem (Life technologies) using a StepOnePlus™ Real-Time PCR System (Applied Biosystems). The sequence of primers were: *β-actin* F : 5'-ATGGAGGGGAATACAGCCC-3', R : 5'‑TTCTTTGCAGCTCCTTCGTT-3', *18S* F : 5’-GTAACCCGTTGAACCCCATT-3’, R : 5’‑CCATCCAATCGGTAGTAGCG-3’, *IL-1β* F : 5’‑GGTCAAAGGTTTGGAAGCAG-3’, R : 5’‑TGTGAAATGCCACCTTTTGA-3’, *NLRP3* F : 5’‑TGTGTGGATCTTTGCTGGG-3’-3', R : 5’-GGAATGTGATGTACACGTGTCATTG-3’. TNF-α F : 5’‑CCACCACGCTCTTCTGTC-3’, R : 5’-CACTTGGTGGTTTGCTAC-3’. Expression was normalized to β-actin and 18S. Relative expression of RNA targets was determined using the comparative ΔΔ*C*_t_ method.

***Lysotracker***

At 4 and 6 hours of different treatments (ctrl, 5‑FU and 5‑FU with DHA or Bafilomycin), adherent MSC-2 cells were labelled with the lysotracker DeepRed probes (Fisher) dissolved at 50 nM in cell culture medium for 30 minutes. Cells were then collected and centrifuged for flow cytometry analysis with BD LSR-II flow cytometer and BD FACSDiva software (BD). Data were analyzed using FlowJo software (Tree Star).

***EL-4 tumor growth, free DHA injections and 5-FU treatment***

Mice (female C57BL/6J, 9 weeks) were subcutaneously injected with 10^6^ EL4 cells and assigned to 5‑FU-non-treated group (ctrl) or 5‑FU‑treated groups. Mice with EL4 tumor (tumor size ~100 mm^2^) received intraperitoneal injections of vehicle or free DHA (30 µg/g body weight; Sigma-Aldrich) 6 days prior to a single intraperitoneal injection of 5‑FU at 50 mg per kg body weight (tumor size ~200 mm^2^). Injections of free DHA or vehicle were performed every two days before and after the single 5‑FU injection. Tumor growth was monitored over the time and tumor surface was calculated according to the formula: length × width.

Animal use and care were approved by the Animal Experiments Ethics Committee of University de Bourgogne (approved protocol #8819).

***Isolation and flow cytometry analysis of MDSC from spleens***

Spleens from EL4 tumor-bearing mice were collected and manually dissociated 48 hours after a single injection of vehicle or 5­‑FU (50 mg/kg). After red blood lysis in lysis buffer (NH_4_Cl 0.83%, KHCO_3_ 0.1%, EDTA 0.1 mM), splenocyte suspension was saturated with mouse FcR blocking reagent (Miltenyi Biotec, Paris, France) and labelled with phycoerythrin-cyanine 7-conjugated anti‑Gr-1 (RB6-8C5, Ozyme, France) and allophycocyanine (APC)-conjugated anti‑CD11b (REA592, Miltenyi Biotec) for 20 minutes at 4°C, dead cells were exclude using 7‑AAD staining. All events were acquired by a BD LSR-II flow cytometer with BD FACSDiva software (BD), and data were analyzed using FlowJo software (Tree Star) to determine the percentage of MDSC in spleen.

***CT-26 tumor growth and MDSC isolation***

For the analysis of pro-IL-1β and NLRP3 mRNA expression in CD11b^+^ Gr-1^+^ cells compared to Gr-1 negative cells, mice (female Balb/c, 9 weeks from Charles River, France) were subcutaneously injected with PBS or CT‑26 cancer cells (500 000 cells). When tumor size reached ~250 mm^2^, spleens were collected from naive and CT-26 tumor‑bearing mice and CD11b^+^ Gr-1^+^ and Gr-1^-^ were purified. Spleens were manually dissociated, red blood cells were lysed (NH_4_Cl 0.83%, KHCO_3_ 0.1%, EDTA 0.1 mM). Cells were saturated by using mouse FcR blocking reagent (Miltenyi Biotec, Paris, France), then Gr-1 fraction was first enriched by using phycoerythrin-cyanine 7 (PE-Cy7)-conjugated antibodies to Gr-1 (RB6-8C5, Biolegend, Ozyme, Montigny-le-Bretonneux, France) and anti‑Cy7 microbeads (Miltenyi Biotec). MDSC were then further sorted as CD11b^+^ Gr1^+^ by using allophycocyanine (APC)-conjugated antibodies to CD11b (REA592, Miltenyi), with a BD ARIA cytometer equipped with BD FACSDiva software (BD Biosciences). Animal use and care were approved by the Animal Experiments Ethics Committee of University de Bourgogne (approved protocol #8821).
