# Supplementary figures and images for: Docosahexaenoic acid inhibits both NLRP3 inflammasome assembly and JNK-mediated mature IL-1β secretion in 5-fluorouracil-treated MDSC: implication in cancer treatment
Source: Cell Death Dis. 2019 Jun 19;10(7):485. doi: 10.1038/s41419-019-1723-x (PMC6584690; doi:10.1038/s41419-019-1723-x)

Supplemental Figure 1

A

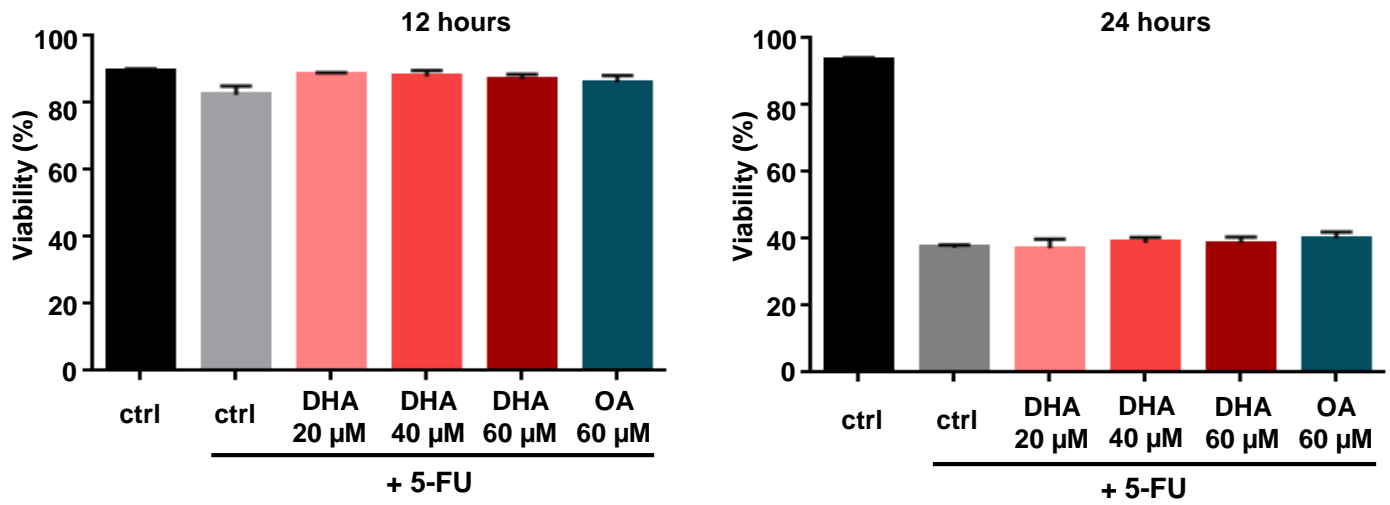

B

C

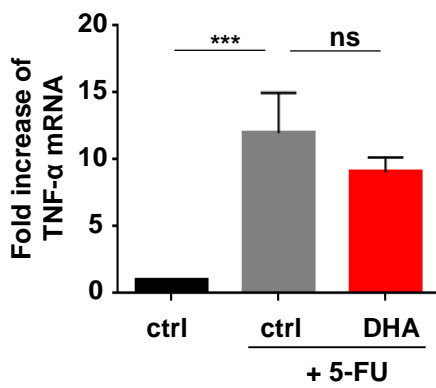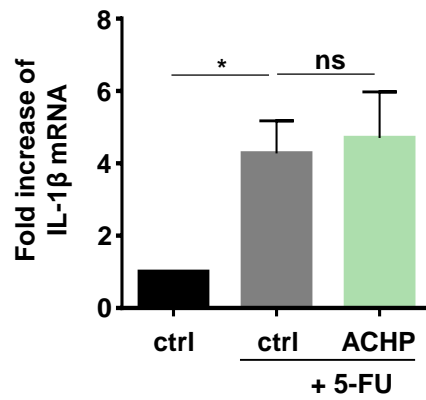

D

E

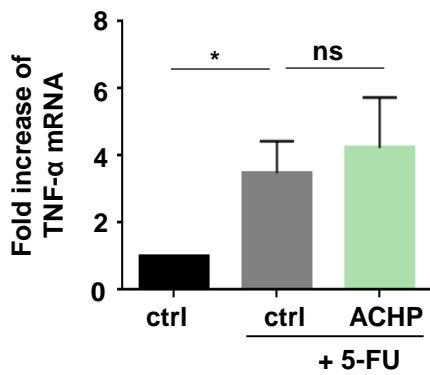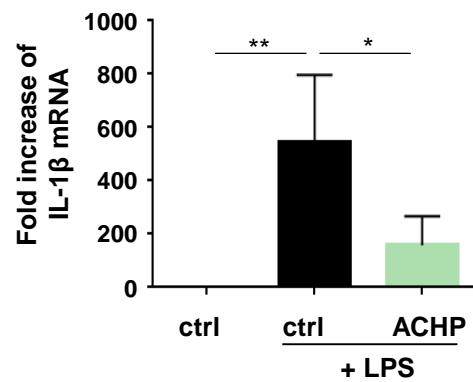

F

G

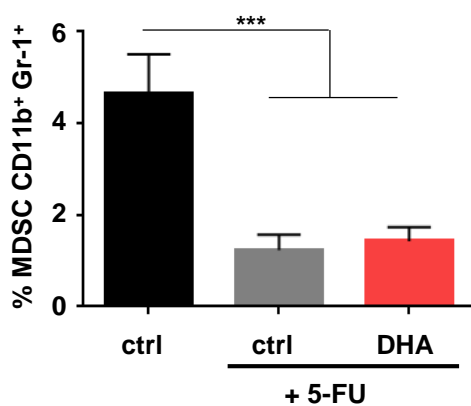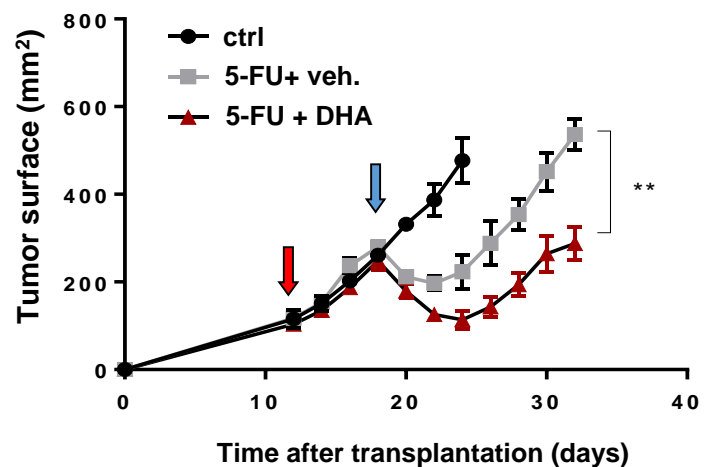

Supplement: Supplementary file 2 — Supplemental Figure 1 [file 41419_2019_1723_MOESM2_ESM.pdf]

Supplemental Figure 2

A

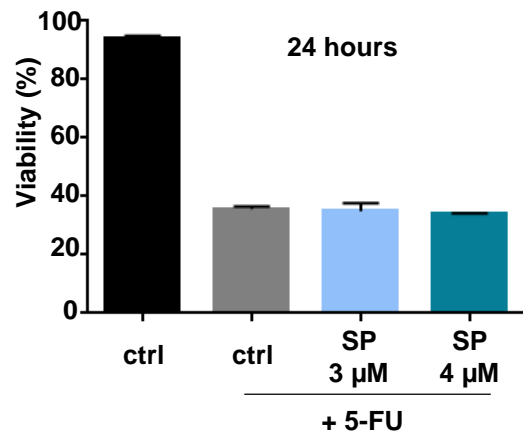

B

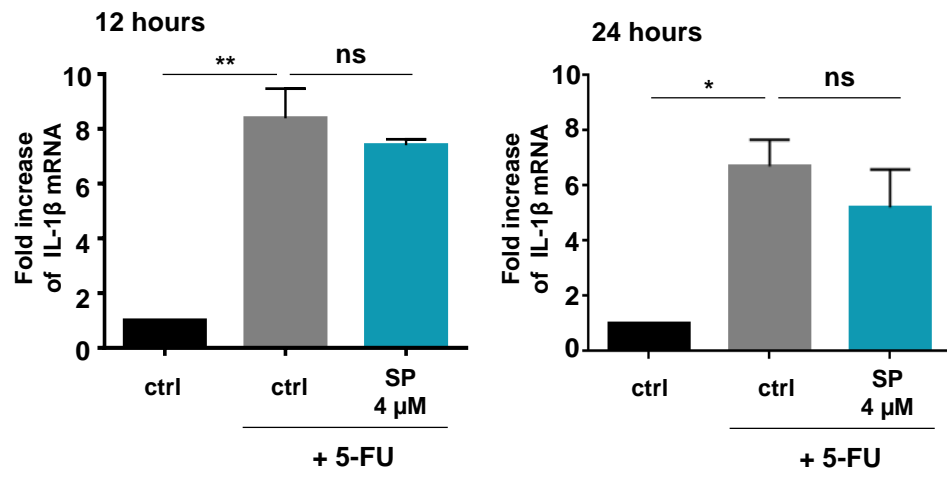

C

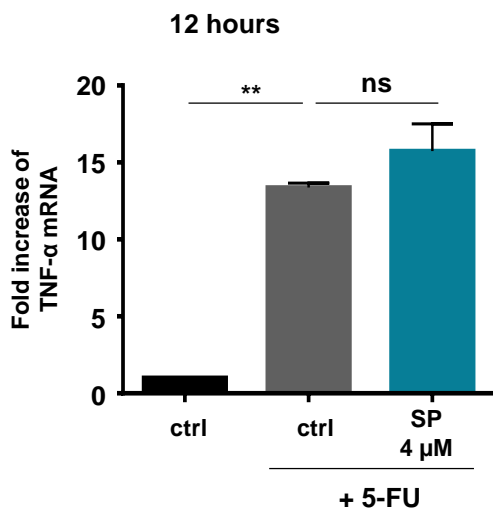

D

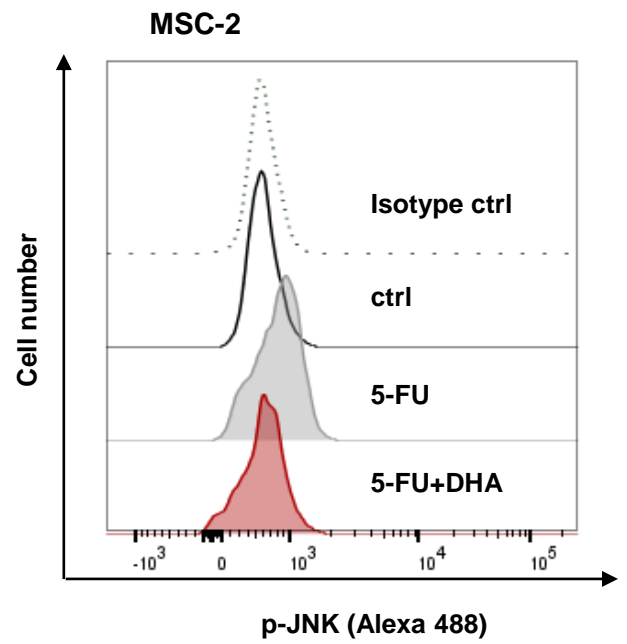

E

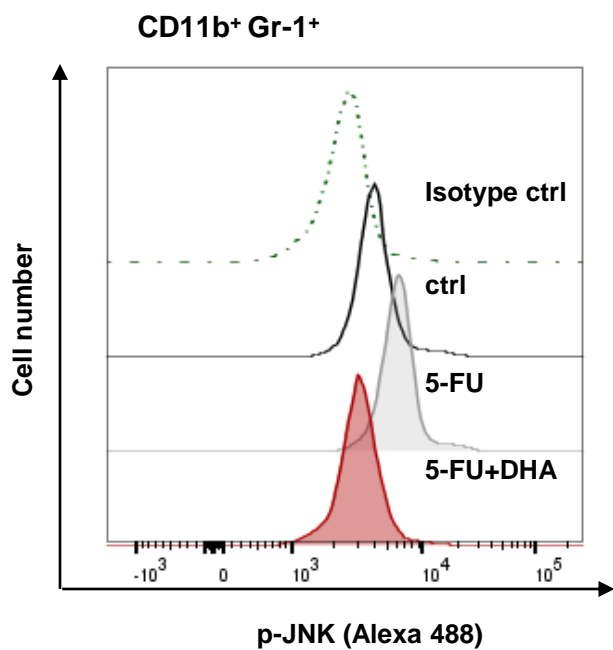

Supplement: Supplementary file 3 — Supplemental Figure 2 [file 41419_2019_1723_MOESM3_ESM.pdf]

**A**

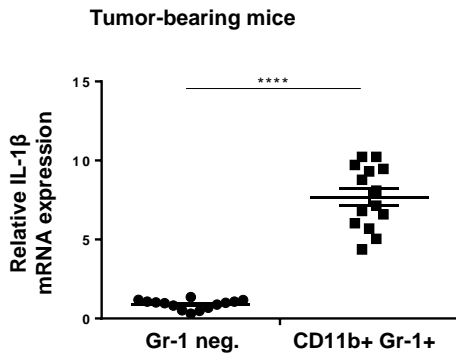

**B**

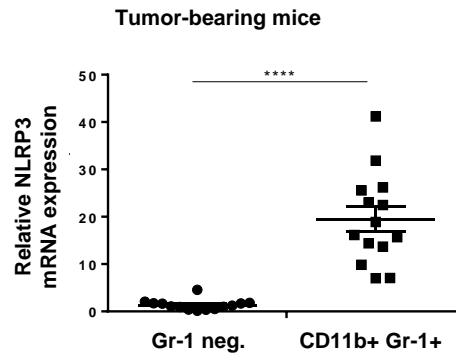

**C**

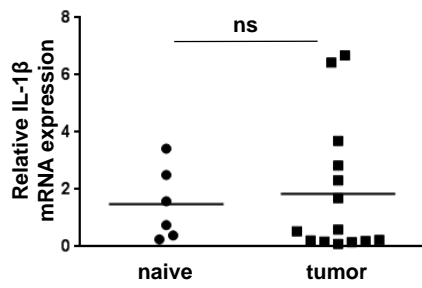

**D**

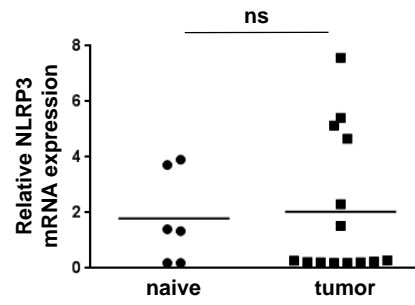

**E**

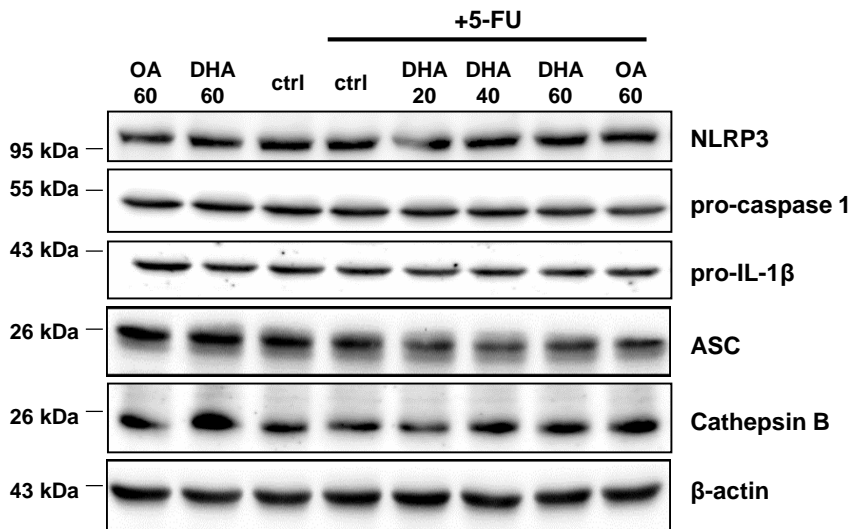

**F**

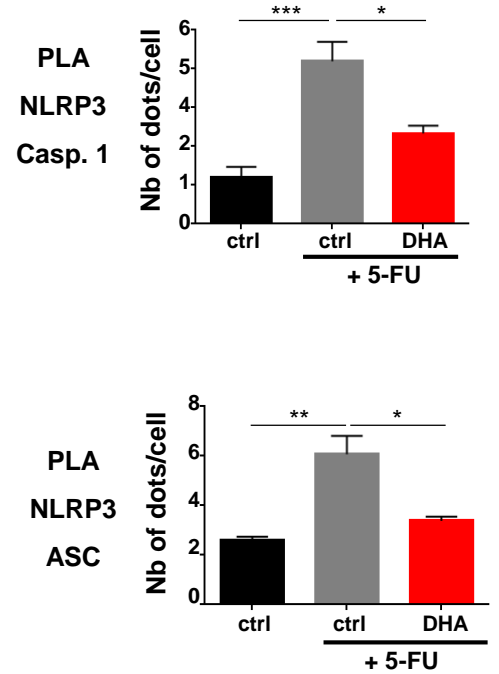

**G**

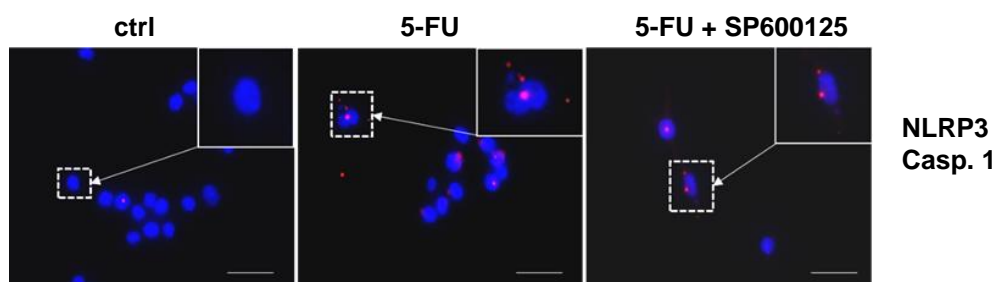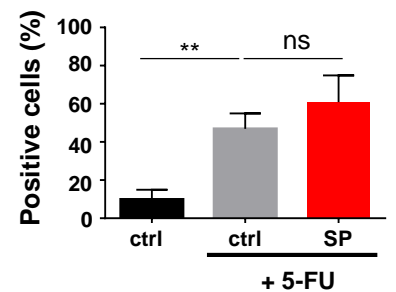

H

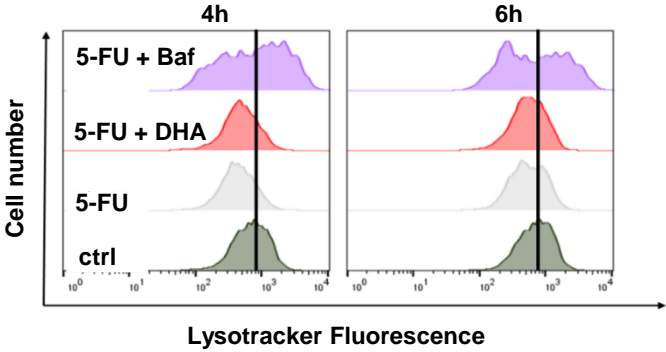

I

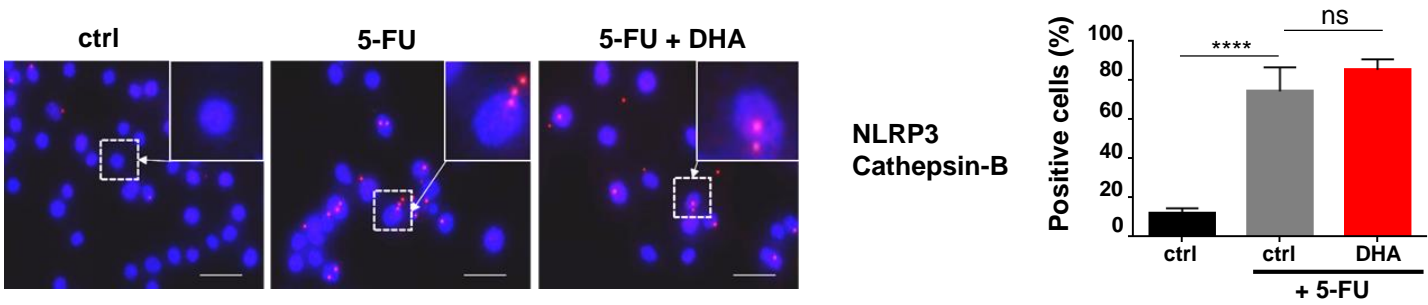

J

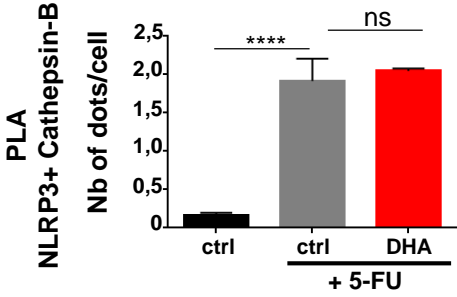

K

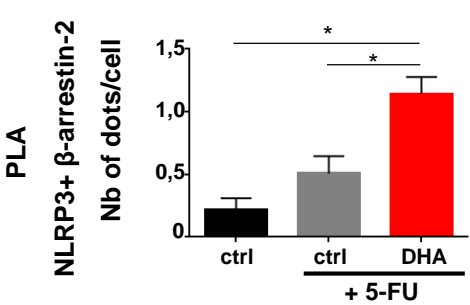

Supplement: Supplementary file 4 — Supplemental Figure 3 [file 41419_2019_1723_MOESM4_ESM.pdf]

A

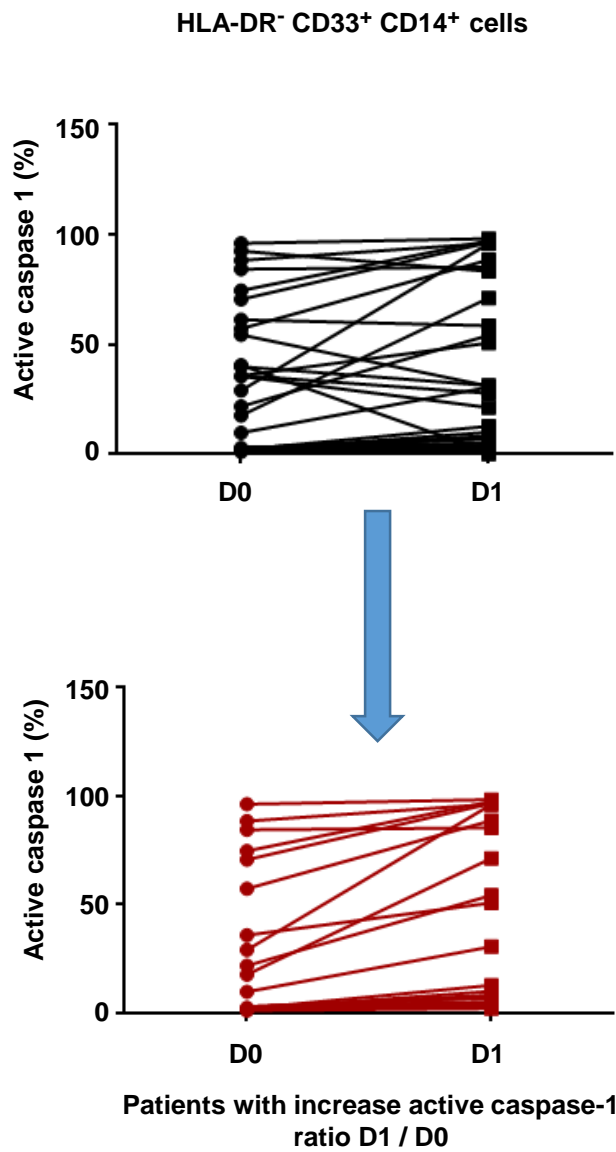

B

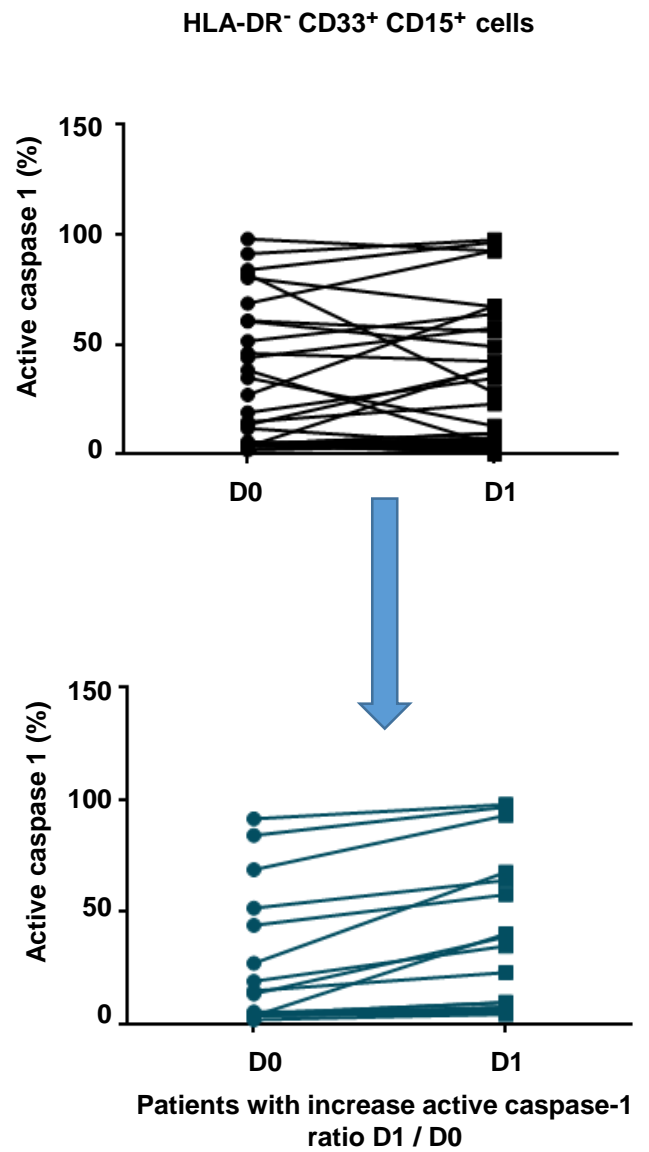

C

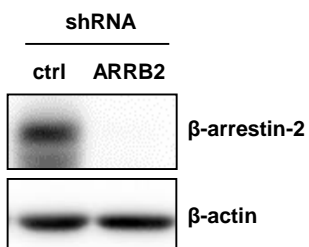

Supplement: Supplementary file 5 — Supplemental Figure 4 [file 41419_2019_1723_MOESM5_ESM.pdf]

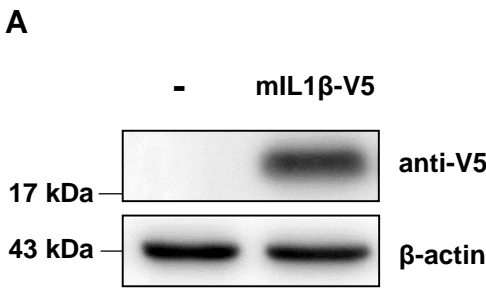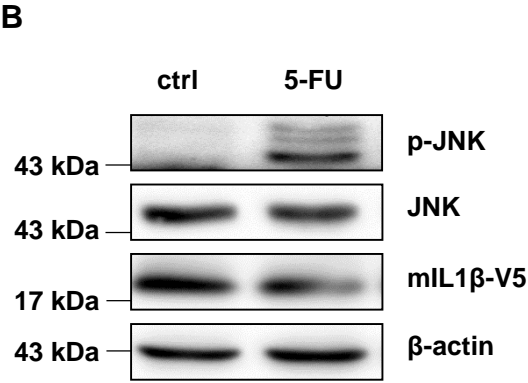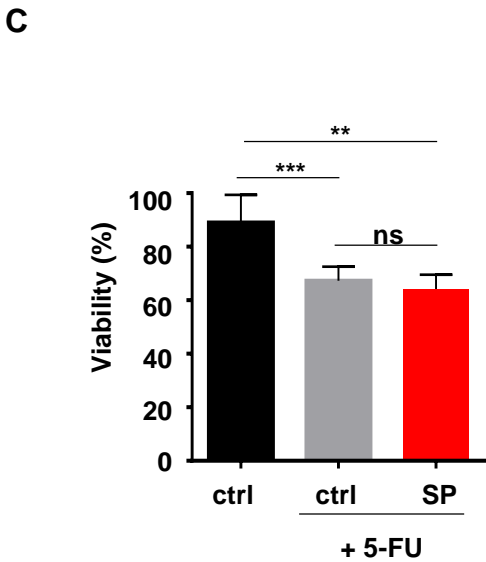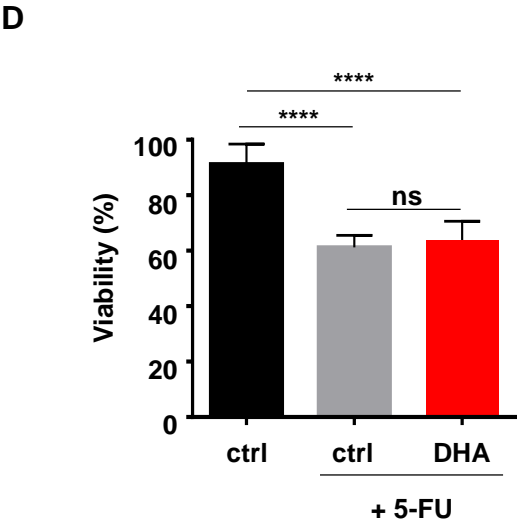

Supplement: Supplementary file 6 — Supplemental Figure 5 [file 41419_2019_1723_MOESM6_ESM.pdf]
